# Atomic resolution of short-range sliding dynamics of thymine DNA glycosylase along DNA minor-groove for lesion recognition

**DOI:** 10.1093/nar/gkaa1252

**Published:** 2021-01-19

**Authors:** Jiaqi Tian, Lingyan Wang, Lin-Tai Da

**Affiliations:** Key Laboratory of Systems Biomedicine (Ministry of Education), Shanghai Center for Systems Biomedicine, Shanghai Jiao Tong University, 800 Dongchuan Road, Shanghai 200240, China; Key Laboratory of Systems Biomedicine (Ministry of Education), Shanghai Center for Systems Biomedicine, Shanghai Jiao Tong University, 800 Dongchuan Road, Shanghai 200240, China; Key Laboratory of Systems Biomedicine (Ministry of Education), Shanghai Center for Systems Biomedicine, Shanghai Jiao Tong University, 800 Dongchuan Road, Shanghai 200240, China

## Abstract

Thymine DNA glycosylase (TDG), as a repair enzyme, plays essential roles in maintaining the genome integrity by correcting several mismatched/damaged nucleobases. TDG acquires an efficient strategy to search for the lesions among a vast number of cognate base pairs. Currently, atomic-level details of how TDG translocates along DNA as it approaches the lesion site and the molecular mechanisms of the interplay between TDG and DNA are still elusive. Here, by constructing the Markov state model based on hundreds of molecular dynamics simulations with an integrated simulation time of ∼25 μs, we reveal the rotation-coupled sliding dynamics of TDG along a 9 bp DNA segment containing one G·T mispair. We find that TDG translocates along DNA at a relatively faster rate when distant from the lesion site, but slows down as it approaches the target, accompanied by deeply penetrating into the minor-groove, opening up the mismatched base pair and significantly sculpturing the DNA shape. Moreover, the electrostatic interactions between TDG and DNA are found to be critical for mediating the TDG translocation. Notably, several uncharacterized TDG residues are identified to take part in regulating the conformational switches of TDG occurred in the site-transfer process, which warrants further experimental validations.

## INTRODUCTION

Thymine DNA glycosylase (TDG), as a repair enzyme that initiates the base excision repair (BER), is responsible for cleaving the glycosidic bond between the mismatched/damaged base and the sugar group (1,2). TDG specifically targets to the G·T and G·U mismatches, and several damaged nucleobases, e.g. 5-hydroxymethyl-U (5hmU), 5-formyluracil (FoU), and 5-halogenated uracil (such as 5FU, 5CIU, 5BrU and 5IU) (3–6). In addition, several chemically modified cytosine rings, including the 5-formylcytosine (5fC) and 5-carboxylcytosine (5caC), can also be corrected by TDG (7,8). These epigenetic modifications have been found to take place in the DNA demethylation process, which plays a profound role in the embryonic development (7,9–11). Therefore, TDG is critical for maintaining the genome integrity, regulating the epigenetic marks, and is also considered as a potential therapeutic target against various diseases, e.g. melanoma and breast cancer (12–14).

As a repair enzyme, TDG requires an efficient strategy to locate the target nucleotides (nts) among billions of cognate ones (15). A widely accepted target-search mechanism for the DNA-binding proteins is described as ‘facilitated diffusion’ model whereby the functional proteins can firstly collide with DNA and establish nonspecific contacts via three-dimensional (3D) diffusion in bulk solution, followed by one-dimensional (1D) sliding (*associative transfer*) or hopping (*dissociative transfer*) searching along the DNA chain to finally locate the lesion sites (15–21). More specifically, the 1D-sliding mode involves loose association of protein and DNA, allowing the protein translocation along DNA and ensuring all base pairs (bps) can be potentially scanned. The sliding dynamics may proceed through various structural orientations of the protein relative to DNA. For example, a simple phosphate tracking mechanism was proposed for uracil DNA glycosylase (UDG) when transferring along a single-stranded DNA chain (22,23). Earlier studies have also indicated that the DNA-binding proteins may rotate along the minor/major groove of DNA through continuously contacting the DNA phosphate backbone or nucleobases (namely, a rotation-coupled sliding motion), e.g. for human 8-oxoguanine DNA glycosylase (hOGG1) (24,25) and lac repressor (26). On the other hand, the 1D-hopping mode can lead to transient dissociation of protein from DNA, then rebinding, thereby bypassing bound obstacles (27,28).

Extensive experimental techniques, including biochemical (23,29–31), single-molecular and florescence approaches (24,25), have been employed to investigate the molecular mechanisms of the 1D target-searching process for various DNA glycosylases. One of the widely studied system is UDG that shares a similar structural fold to TDG (32). In particular, by employing an UDG inhibitor, uracil, as a molecular-lock, Stivers *et al.* were able to trap the unbound UDG as it transiently dissociates from DNA, thereby the dissociative and associative transfer event can be differentiated. Their results suggest that the associative transfer distance for UDG is less than 10 bp at low ion concentration (23), and is not over 40 bps within cellular environment (33). In addition, the dissociation event in human cell can occur at least once when UDG diffuses at a distance > 40 bps (33). Another extensively investigated DNA glycosylase is hOGG1 that is responsible for excising the damaged guanine, namely 8-oxoguanine. Former single-molecular fluorescence work suggests a rotation-coupled sliding movement of hOGG1 along DNA by rotating along the DNA helix and continuously contacting the DNA phosphate backbone or nucleobases (24,25). Recent biochemical studies, however, indicate the concurrence of both dissociative and associative transfers for hOGG1, with associative sliding distance less than 40 bps (30). Likewise, O’Brien's group found that alkyladenine DNA glycosylase (AAG) is also capable of translocating along DNA at a short distance, whereas the hopping mode likely dominates the searching process for longer distances (34). Moreover, crowding environments can substantially increase the possibility of the associative transfer for DNA glycosylases (21,33,35). Taken together, all the aforementioned DNA glycosylases can undergo a short-range (a few bps) associative transfer along DNA, although they exhibit distinct structural folds.

Since TDG and UDG belong to the same structural family (32), it is highly plausible that TDG may also employ a similar sliding mechanism to UDG for target search when approaching to the lesion site. Previous X-ray crystallographic studies have obtained one static structure of non-specific TDG–DNA complex where the interrogated G·C bp is non-flipped (PDB id: 2rba) (36). In addition, recent atomic force microscopy (AFM) and fluorescence studies found that the TDG binding can induce significant DNA bending for both specific and non-specific chains (37). Despite the above efforts, atomic-level observation of the 1D-sliding dynamics of TDG along DNA is still challenging for current experimental techniques due to the limited spatial-temporal resolution. Computational simulations, on the other hand, can provide an atomic-level understanding of both structural and kinetic properties for almost all the critical biomolecules in the living system (38,39). More importantly, to overcome insufficient sampling problems suffered by conventional molecular dynamics (MD) simulations, one can now construct a Markov state model (MSM) to investigate the structural dynamics of biomolecules occurred at relatively longer timescales (i.e. hundreds of microsecond (μs) or even longer), through integrating hundreds of short-time MD simulations (i.e. hundreds of ns) (40–45).

In details, by coarse-graining the phase space, the conformations sampled from the raw MD simulations can be firstly grouped into hundreds of microstates according to certain distance metric, such as the geometric features. Then, one can construct a transition probability matrix **T** (TPM) in which each entry *T_ij_* represents the transition probability for the *i* → *j* transition under a certain lag time *τ*. The chosen *τ* should be long enough to ensure that all the conformations within any microstate can be well equilibrated (to avoid the internal barrier). The markovian property can be satisfied only if for any transition *i* → *j*, the state *j* is only depend on state *i* but not the preceding states. In this case, one can propagate the conformational dynamics to any timescale of interest using the following equation:

$$\begin{equation*}{\rm{P}}\left( {n\Delta t} \right) = {\left[ {{\rm{T}}\left( {\Delta t} \right)} \right]^{\rm{n}}}{\rm{P}}\left( 0 \right)\end{equation*}$$

where P(0) and P(nΔ*t*) represent the state populations at time 0 and *n*Δ*t*, respectively. Both thermodynamic and kinetic properties can then be readily obtained by resolving the eigenfunctions of the matrix **T** (46). Until now, the MSM method has been employed to investigate many biological systems (45,47–56), including TDG (57–59).

Here, by constructing MSMs based on 252 100-ns MD simulations (integrated simulation time of ∼25 μs), we reveal, at atomic resolution, the short-range sliding dynamics of TDG along DNA minor-groove for lesion search and recognition. By employing a G·T-mispair containing DNA chain, we captured several key metastable states during the TDG sliding and deciphered the detailed mechanisms of how TDG locates the lesion-site, sculptures the DNA backbone and finally invades the DNA minor-groove. The functional roles of the electrostatic interactions in mediating the site-transfer of TDG between different bp sites were also carefully examined. Our work provides deep structural insights for the target-searching mechanism of TDG at atomic details and warrants further experimental studies.

## MATERIALS AND METHODS

### Constructing various TDG–DNA complexes with TDG bound at different bp sites

**Figure 1.**
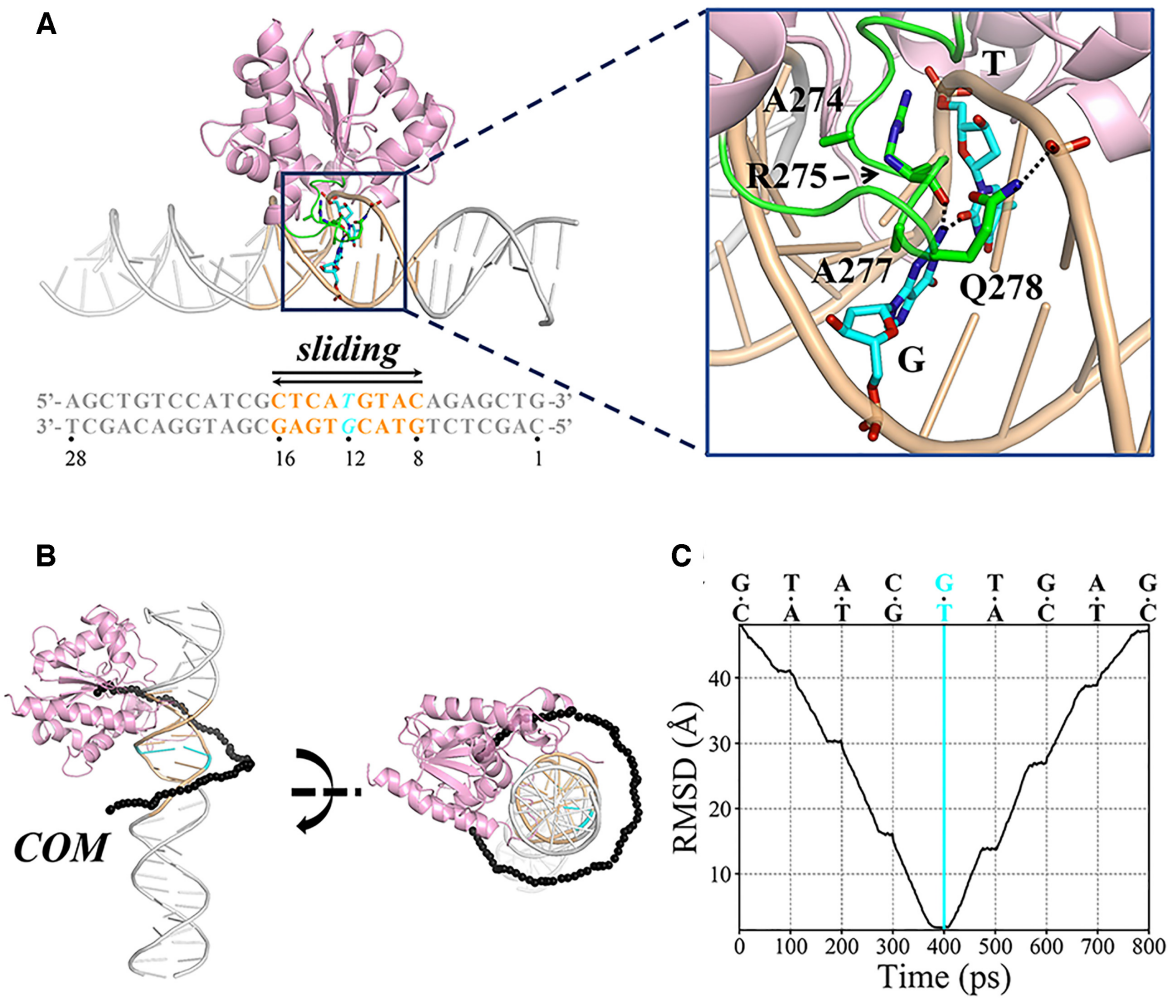
(**A**) The modeled TDG–DNA complex with TDG targeting to the G·T mispair. The intercalation loop (P270–R281) of TDG is colored in green and the other parts are colored in pink. The DNA chain is shown in gray, with the G·T mispair and the TDG-sliding region highlighted in cyan and orange color, respectively. The DNA sequence used in this study is provided below with each bp labeled. The key residues surrounding the target site are highlighted in sticks and a zoomed-in view is provided on the right panel. The hydrogen bonds (HBs) are shown in black dashed line. (**B**) Initial TDG-sliding pathway along DNA obtained by TMD simulations. The center of masses (COMs) of representative TDG structures along the sliding pathway are shown with black spheres in two different views. (**C**) RMSD (root-mean-squared deviation) of the C_α_ atoms of TDG during the TMD simulations for the forward direction (from bp 8 to 16), with respect to the lesion-targeting TDG–DNA IC (at bp 12). Each interrogated bp is shown on the top.

To study the sliding dynamics of TDG along DNA that contains one G·T mispair, we constructed nine initial TDG–DNA interrogation complexes (ICs) prior to base-flipping, for each, TDG interrogates one certain bp site. To achieve this, we firstly modeled a B-form DNA chain containing 28 bps, using the same sequence adopted from one crystal structure of TDG (PDB id: 5hf7) (60) and substituted the U^F^ with T to form the G·T mismatch bp (see Figure 1A for the DNA sequence). Notably, former structural and AFM studies have observed that the TDG binding can induce a profound DNA bending prior to base-flipping, with a bend angle of ∼30° (36,37). We therefore created a 30° bending form of the above B-form DNA by modifying the roll angle between the G·T mispair and its adjacent C·G bp. Next, we built a TDG–DNA complex with TDG targeting to the intrahelical G·T mispair based on the above 30° B-form DNA and two TDG crystal structures (PDB id: 5hf7 and 2rba) (36,60). In details, we first constructed a TDG–DNA IC as described in our previous study, including the TDG residues K107 to E303 (58), and then by superimposing the sugar heavy atoms of the G·T mispair and one adjacent T·A bp between the above modeled IC and the 30° B-form DNA to finally generate a new TDG–DNA IC in which the TDG structure is derived from 5hf7 and DNA is a 30° bended B-form. Since the intercalation loop from 5hf7 is in a penetration form, we then extracted the non-specific loop-conformation from 2rba (residues S273 to R281) to replace the counterpart region in 5hf7. Finally, the modeled TDG–DNA IC was subject to energy minimization (see Figure 1A).

Based on the above lesion-targeting IC, we constructed eight additional TDG–DNA ICs where TDG interrogates varied bp sites flanking the G·T mispair (at the bp 8–11 and 13–16, see Figure 1A). In specific, for each given bp site, we used the same modeling strategy as described above. That is, by superimposing the sugar heavy atoms of the given bp and one adjacent bp of the 30° B-form DNA to the counterpart groups of the G·T mispair and its adjacent T·A bp in the lesion-targeting IC, we can then construct a new IC in which TDG interrogates the given bp site. Thus, we finally obtained a total of nine TDG–DNA ICs with TDG interrogating nine consecutive bp sites (from bp 8 to 16), and for each IC, energy minimization was performed (see Figure 1A and Supplementary Figure S1). The 9 ICs were finally served as the input structures for the following target molecular dynamics (TMD) simulations.

### Obtaining the initial TDG sliding path along DNA by TMD simulations

Based on the above 9 TDG–DNA ICs, we derived an initial sliding path of TDG along DNA by performing a total of 8 TMD simulations. All the TMD simulations were performed using the AMBER14 software (61), and the TDG–DNA structures were described by the amber force field ff99SB with torsional corrections for the nucleic acids (62–65). For each TMD simulation, two TDG–DNA complexes where TDG interrogates two consecutive bp sites were employed as the initial and target structures, respectively. Each simulation time was set to 100 ps, during which an exerted force was applied to restrain the DNA backbone and ribose heavy atoms, with a force constant of 10 kcal/mol/Å^2^. The targeting region was set as all the TDG C_α_ atoms, upon which a pulling force was used to drive TDG moving from the initial bp site to the target site, with a pulling force constant of 0.5 kcal/mol/Å^2^. Finally, by integrating eight separate TMD trajectories, we obtained a complete sliding path of TDG along a 9-bp DNA segment (from bp 8 to 16, see Figure 1A). In addition, the same strategy was applied to the reverse direction, namely from bp 16 to 8. We thus collected a total of sixteen 100-ps TMD trajectories, and obtained two sliding paths of TDG in opposite directions (see Figure 1B, C and Supplementary Figure S2).

Next, to choose the input structures for the subsequent enhanced samplings, we performed geometric clustering based on the above two sliding paths of TDG. The geometric clustering was performed by firstly decomposing the high-dimensional conformational dataset onto the top four principal vectors using the time-structure-independent component analysis (tICA) (66–68) implemented in the MSMbuilder 3.8 package (42). We selected a total of 1314 distance pairs between the following atoms as the input parameters for the tICA projection (termed as dp1 in Supplementary Figure S3):

The P atoms of the DNA segment (bp 8–16)—C_α_ atoms of several TDG motifs (including K107-K122, P141-H158, T196-D202 and P270-A282).

The P atoms of the DNA segment (bp 8–16)—side-chain heavy atoms of the TDG residues R275, Q278 and F279.

We then further clustered the projected dataset into 90 classes by the *k-centers* method. Finally, we chose the central structure for each of the above 90 classes as the representative structure for the following unbiased MD simulations.

### Shooting extensive unbiased MD simulations

We performed a total of two rounds of unbiased MD simulations using the Gromacs package (69). In the first round, we performed 90 20-ns MD simulations starting from each of the above selected structures derived from the TMD simulations. The force fields used for the TDG and DNA are the same as that used in the TMD simulations. Each TDG–DNA structure was firstly infiltrated in a triclinic box filled with 24 904 SPC water molecules (70). To neutralize the system and ensure an ionic concentration of 0.15 M, 119 Na^+^ and 70 Cl^−^ were added to each box. The cutoff distances for the non-bonded terms (van der Waals and short-range electrostatic interactions) were set to 12 Å; the Particle-Mesh Ewald (PME) method was used to calculate the long-range electrostatic interaction (71); the LINCS algorithm was used to constrain all the chemical bonds (72). The final system contains a total of 79 840 atoms. We then carried out energy minimization using the steepest decent method, followed by a 200-ps NVT MD simulation by constraining all the heavy atoms of the TDG–DNA complex. Next, each system was gradually heated from 50 to 310 K within 200 ps and maintained at 310 K using the velocity rescaling thermostat (73). Finally, we performed a 20-ns NVT MD simulation at 310 K for each system by constraining four terminal P atoms of each DNA end.

We then performed geometric clustering for the TDG–DNA conformations obtained from the first round of MD simulations. To eliminate the exerted biases introduced by the TMD simulations, we only retained the last 10-ns dataset of each 20-ns MD simulation for the clustering. The clustering was conducted using the same strategy as described above, namely, dimension decomposition using tICA followed by *K-centers* clustering for the low-dimensional dataset. The 90 clusters were finally obtained and each cluster center was used as the input structure for the second round of MD simulations. For each center structure, we performed three parallel 100-ns MD simulations with varied initial velocities, using the same MD parameters as described above. We finally collected a total of 252 100-ns MD trajectories with an aggregated simulations time of ∼25 μs for the MSM construction.

### MSM construction and validation

According to the TPM, the implied timescale can be calculated by solving the following equation:

$$\begin{equation*}{\tau _k}=-{\tau}/ {{\ln {\mu_k}(\tau)}}\end{equation*}$$

where *τ* represents the lag-time used for building the TPM; *μ_k_* represents the *k*th eigenvalue of the TPM under a lag time of *τ*. By varying the lag-time *τ*, one can obtain the corresponding implied timescale plot, and if the curves start to level off, suggesting that the system is memoryless (Markovian property). Notably, each implied timescale curve represents the average transition time between two sets of groups, one can therefore estimate the timescale for the rate-limiting step involved in the structural dynamics of interest (74). Here, we used a splitting and lumping strategy to construct the MSM. We firstly examined whether different sets of input distance pairs might affect the slowest implied timescale obtained from the MSM. In addition to the dp1 described above, the dp2–7 were designed to evaluate whether inclusion or exclusion of any TDG motif might impose any influence on the slowest implied timescale (see Supplementary Figure S3). In dp2, the TDG motif K232-F243 was included; in dp3, three TDG residues R275, Q278 and F279 were excluded; in dp4–7, the DNA-binding TDG motifs used in dp1 was individually excluded.

Recently, a generalized matrix Rayleigh quotient (GMRQ) was introduced to capture the slow dynamical modes involved in the system of interest (42,75,76). We then firstly validated the selection of the input distance pairs used for the MSM construction by conducting the GMRQ test for the slowest timescale using a tICA correlation lag-time of 20 ns, and cross-validated using a training/test ratio of 1:1 (each for 50 random trials). The results show that the removal of the key intercalation-loop region (namely dp5) is unexpectedly found to perform the best in capturing the slowest dynamics comparing to other sets of distance pairs (see Supplementary Figure S4), which is apparently contradict with the sense that the intercalation-loop must play an essential role in targeting to the lesion sites by inserting into the DNA minor-groove. As a comparison, we plotted the implied timescale curves for each set of input distance pairs with estimated standard errors. The results show that exclusion of any DNA-binding motif can result in faster implied timescales compared to the model constructed using dp1, also for the three key TDG residues (see Supplementary Figure S5). Moreover, inclusion of additional TDG motif can barely impact the slowest implied timescale (see Supplementary Figure S5), We thus conclude that the dp1 is sufficient to describe the principal conformational dynamics for the TDG searching process.

The failure of the GMRQ test with cross-validation for our simulation dataset is likely because that the TDG searching dynamic studied here is a sequential transition process (see the Results section). Therefore, the training and test sets with each accounting for only half of the complete dataset could likely lead to low-connectivity of the state-to-state transition due to the exclusion of the critical transition event observed in certain MD trajectories. Therefore, the GMRQ validation method is unreliable for our current MD samplings.

Therefore, to examine the effects of various hyperparameters used for the MSM construction on the slowest dynamics, we projected MD conformations onto four slowest tICs using dp1, and then applied the K-centers algorithm to cluster the low-dimensional conformations into different number of states (500, 600, 700 and 800 states respectively) and also under different tICA correlation lag-time (10, 20 and 30 ns, respectively). The implied timescale plots for each of the above 12 models are shown in Supplementary Figure S6. The results demonstrate that the MSM constructed using a correlation lag time of 20 or 30 ns displays better Markovian properties than that built at the correlation lag-time of 10 ns owning to the well-converged curves. The microstate number, on the other hand, imposes inconsiderable influence on the slowest implied timescale (all converged at ∼hundreds of μs).

We finally evaluated the effects of the tIC number on the slowest dynamics by constructing five MSMs using different number of tICs, namely tIC1–5. The results indicate that using tIC1 alone can lead to a relatively faster implied timescale, whereas inclusions of tIC2–4 give rise to comparable and converged implied timescale curves (∼hundreds of μs, see Supplementary Figure S7A–D). The top five tICs, on the other hand, would get the Markovian property worse, likely due to the low connectivity of the transition matrix (see Supplementary Figure S7E). This result suggests that the top 4 tICs are reliable to describe the conformational dynamics involved in the system. Therefore, we finally chose the state number of 500, tICA correlation lag-time of 20 ns and top four tICs for the final MSM construction. Then, to visualize the key intermediate states involved in the sliding dynamics, we further lumped the 500-state kinetic model into 9 macrostates by the PCCA+ algorithm (77) implemented in the MSMbuilder 3.8 package (42).

We finally performed convergence test to ensure our MD sampling is sufficient to construct a reliable MSM. In specific, we truncated each 100-ns MD trajectory into 4 sub-datasets, namely 0–70, 0–80, 0–90 and 0–100 ns, respectively. We thus collected 4 datasets with different aggregated simulations time, each containing 18 μs (70 ns × 252), 20 μs (80 ns × 252), 23 μs (90 ns × 252) and 25 μs (100 ns × 252), respectively. Based on each dataset, we constructed several MSMs by varying the lag-time and calculated the corresponding implied timescale for each MSM. The results show that the slowest implied timescales are all converged to hundreds of μs (see Supplementary Figure S8), and all curves tend to level off after a lag-time of 30 ns, we thus chose the lag-time of 30 ns for the calculations of the thermodynamic/kinetic properties. In addition, we further projected each dataset onto the same two slowest tICs. The free energy landscapes show that no additional metastable state appears when the simulation time increases (see Supplementary Figure S9). The above analyses indicate that our MD dataset are sufficient to build a reliable MSM.

### Calculating the mean first passage time (MFPT) and the stationary distributions

We finally calculated the MFPT between each pair of macrostates and the stationary distribution of each state. We first generated a 10-ms long Monte Carlo (MC) trajectory according to the TPM built with 500 microstates using a lag-time of 30 ns. The MC trajectory is long enough to ensure well-equilibrated transitions between different states, so that the MFPT and stationary populations can be readily obtained. To estimate the corresponding standard error, we generated 100 trajectory lists, each containing 252 randomly selected trajectories from the original MD simulations. For each trajectory list, we generated a new 10-ms MC trajectory and calculated the corresponding MFPT and stationary populations. Finally, the mean values were averaged over the 100 datasets and the corresponding standard errors were calculated.

## RESULTS

### Exploring the conformational space for TDG sliding process along DNA

To investigate the molecular mechanisms underlying the sliding dynamics of TDG along DNA at a searching distance ∼9 bp, we firstly constructed nine TDG–DNA complexes based on a G·T-mispair containing DNA duplex with a 28-bp length. For each complex, TDG interrogates different bp sites spanning the bps from 8 to 16 (see Figure 1A and Supplementary Figure S1), in particular, the G·T mismatch locates in the middle (at bp 12). The constructed complexes were then subject to energy minimization (see MATERIALS AND METHODS for the details of the model construction). Notably, each above TDG–DNA complex is an interrogation complex (IC) where the inspected bp is non-flipped and the key intercalated residue R275 is lying along the minor groove rather than penetrating into the base stack (see Figure 1A and Supplementary Figure S1 for the energy minimized structures). In specific, for the lesion-targeting IC (at bp 12), one structural motif from the intercalation loop, consisting of A274-A277, can stretch into the DNA minor-groove. Particularly, the A277 sidechain forms hydrophobic contacts with the opposing G of the mismatched T; the R275 mainchain can establish one hydrogen bond (HB) with the G12-base; the R275 sidechain electrostatically interacts with the phosphate group of the mismatched T, and Q278 sidechain forms one HB with the DNA backbone (see Figure 1A). In addition, the G·T mispair slightly opens up and the wobble HBs are not ideally formed (see Figure 1A). Then, starting from the above nine TDG–DNA ICs, we derived initial sliding dynamics of TDG along DNA in two opposite directions by performing TMD simulations. To extensively explore the conformational space of TDG along the above sliding paths, we collected a MD simulation dataset with an integrated simulation time of ∼25 μs and finally constructed an MSM to reveal the critical intermediates involved in the TDG translocation and obtained the corresponding thermodynamic and kinetic properties (see Materials and Methods for the details of the TMD and MD setups).

Finally, we examined to what extent the MSM results are biased by the initial TMD simulations. We projected the conformations derived from the TMD and following unbiased MD simulations onto the same top three eigenvectors obtained by the *Isomap* algorithm that is a nonlinear dimensionality reduction technique (78). The results show that the MSM samplings could profoundly deviate from the initial TMD pathways (see Supplementary Figure S10). We thus believe that our extensive unbiased MD simulations can effectively eliminate the bias introduced by TMD simulations.

### TDG targets to the lesion site via inserting the intercalation loop deeply into the minor groove and sculpturing DNA shape

**Figure 2.**
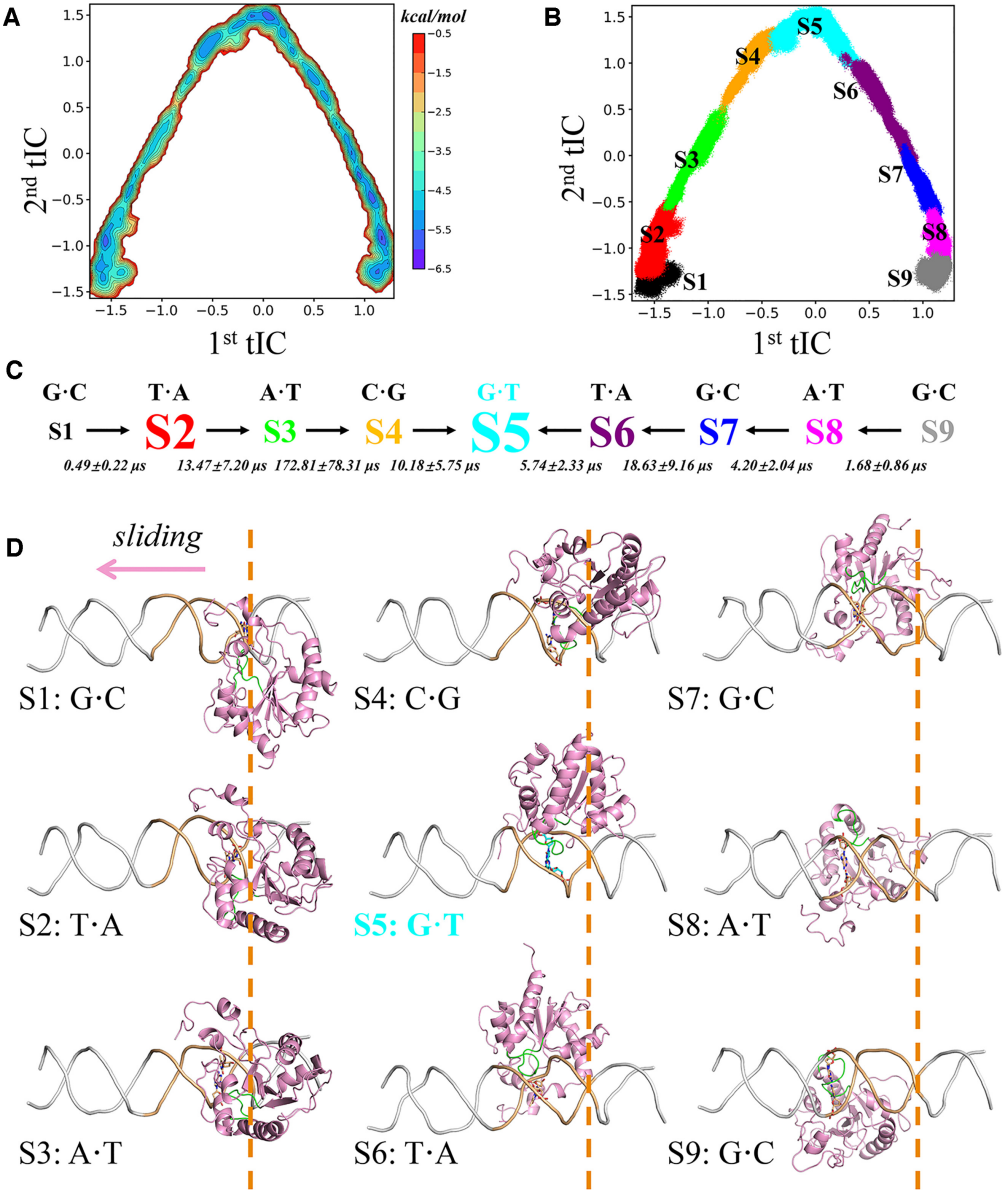
MSM identifies nine metastable states during the TDG translocation along DNA via a rotation-coupled sliding mode. (**A**) Free energy profile for all the MD conformations projected onto the top two slowest tICs. (**B**) The scatter plot of all MD conformation mapped onto the same tICs in A, with each state colored and labeled. (**C**) The nine-state kinetic model derived from the MSM, and the font size roughly reflects the equilibrium population 4.1 ± 2.1% (S1), 15.3 ± 5.5% (S2), 8.8 ± 3.3% (S3), 10.5 ± 3.0% (S4), 18.4 ± 4.3% (S5), 12.7 ± 2.9% (S6), 11.1 ± 2.7% (S7), 11.0 ± 2.7% (S8), 8.1 ± 3.0% (S9). The MFPTs for the inter-state transitions are also provided. Two major searching pathways determined by TPT are indicated by black arrows. (**D**) Representative structures for nine macrostates. Each structure is randomly selected from the most populated microstate of each macrostate, with the interrogated bp-position for S1 indicated by orange dashed lines.

**Figure 3.**
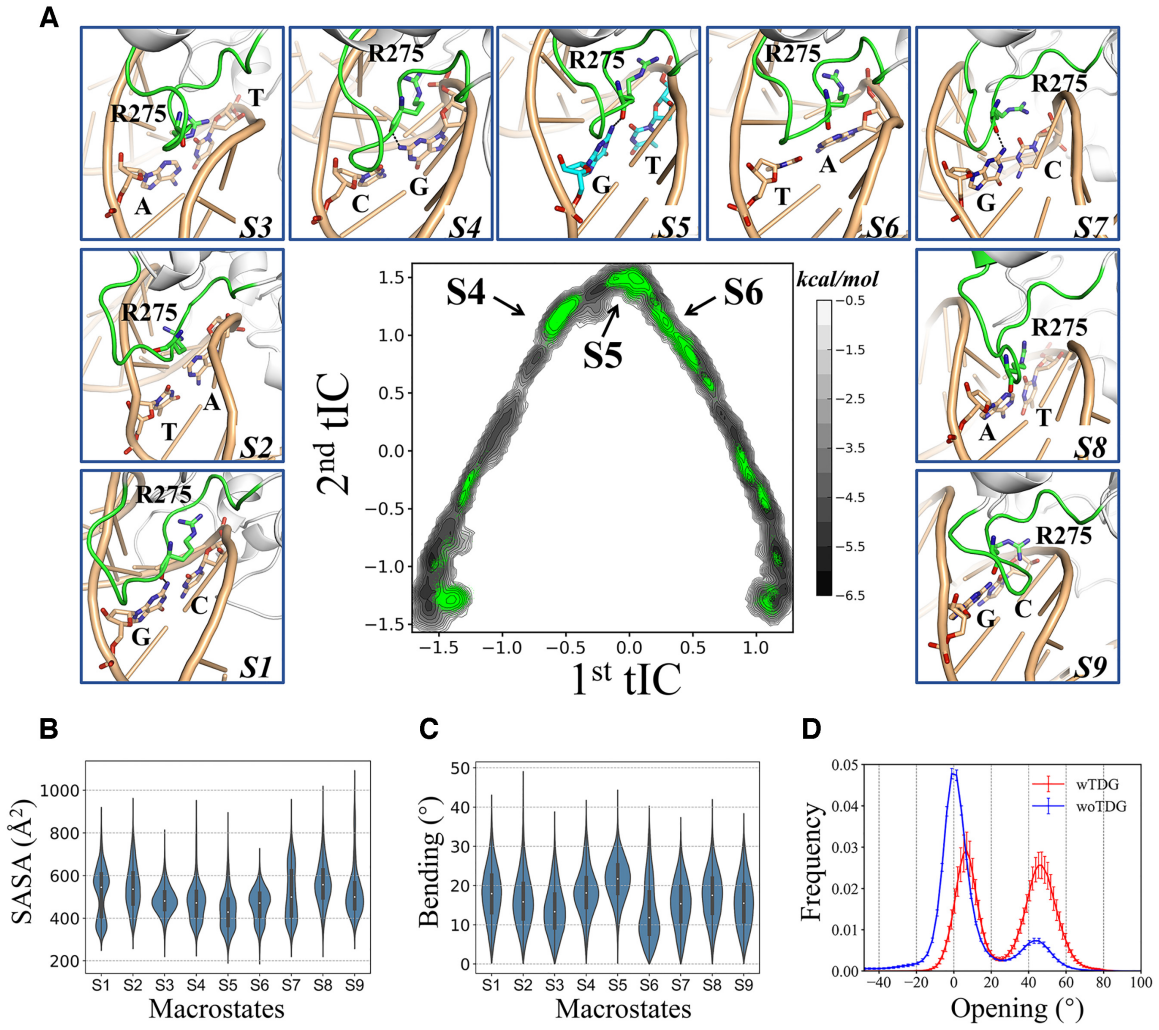
TDG targets to the lesion site via inserting the intercalation loop deeply into the minor groove, resulting in a specific loop and DNA conformations. (**A**) Highlights of the structural features of the intercalation loop for each macrostate (S1–S9), the same conformations from Figure 2D are used. The key residues are highlighted in sticks, and HBs are shown with black dashed lines. See Figure 1 for more details of the structural representations. (*Middle panel)* Scatter plot of the MD conformations projected onto the same two tICs as shown in Figure 2A. For the MD conformations shown with green dots, the intercalation loop displays a crystal-like conformation, with a RMSD < 3 Å. The RMSDs were calculated by fitting the ribose heavy atoms (the interrogated bp and its adjacent bp) to the corresponding IC. The free energy landscape shown in Figure 2A is also provided here as a background reference (shown in black-gray scale). (**B**) Violin plots of the solvent accessible surface area (SASA) of the intercalation loop for each state. (**C**) The DNA bending angle at the G·T mismatched site for each state. Refer to Supplementary Figure S13A for the definition of DNA bending. (**D**) Distribution of the opening angle for the G·T mispair when TDG targets to the lesion-site (wTDG, red line) or other sites (woTDG, blue line). Refer to Supplementary Figure S13B for the definition of opening angle, which is calculated using the Curves+ program (89). The errors of the opening angles were estimated using the bootstrapping strategy, by randomly choosing 252 MD trajectories from the original MD simulations for 50 times. For the calculation of SASA and DNA bending, all the value belonging to the same macrostate are included, with the medians indicated by white circles.

Our MSM reveals nine metastable states during the sliding dynamics of TDG along the DNA chain, namely S1–S9. For each state, TDG interrogates a certain bp site, complying with the idea that it is thermodynamically more favorable when TDG interrogates the bp site than the situation that TDG deviates from the bp site, i.e. the transition state between two adjacent bps. Notably, we observed a strong correlation between the longitudinal and rotational motions of TDG with respect to DNA, suggesting that the TDG translocation roughly follows a rotation-coupled sliding path along the DNA helix (see Supplementary Figure S11). It is also noteworthy that our 100-ns MD simulations are still too short to observe any huge structural changes of TDG that can significantly deviate from the initial paths, such as the complete dissociation of TDG from DNA. Nevertheless, we indeed captured some TDG conformations that exhibit weak salt-bridge interactions with DNA backbones (see followings). In details, the S5 state is determined to be the specific IC and others can be assigned as non-specific ones (see Figures 2 and 3A). As expected, the dominant lesion-searching pathways, determined by the transition path theory (TPT) (79–81), follow two major transition paths, namely, S1→S2→S3→S4→S5 and S9→S8→S7→S6→S5 (see Figure 2C and D). Notably, S5 is determined to be thermodynamically most stable among all states. Moreover, according to the MFPT results, the timescale for the state-to-state transition ranges from few to hundreds of μs, and the rate-limiting step for the above two searching paths occurs in the S3→S4 and S7→S6 transition, respectively. In comparison, the transitions that are distant from the lesion site, e.g. the S1→S2 and S9→S8, take place at a relatively faster rate (see Figure 2C & D, and Supplementary Figure S12B for the MFPTs in the backward transitions).

The intercalation loop in TDG is considered to play critical roles in target recognition and base-flipping process (36,57,58). Here, we find that the intercalation loop can adopt a variety of distinct conformations as TDG slides along DNA, and targeting to the lesion site can result in deep insertion of the intercalation loop into the minor-groove accompanied with solvent expulsions. As shown in Figure 3A, the representative structures for the nine metastable states indicate that the intercalation loop displays distinct conformations and penetrates into the minor groove in varied extents (see Figure 3A and Supplementary Figure S12A). Notably, in the nine minimized ICs (as shown in Supplementary Figure S1), the intercalation loop adopts a conformation ready for the DNA penetration by forming direct contacts with the interrogated bp. We therefore used these ICs as reference structures to quantitatively measure to what extent the intercalation loop would penetrate into the DNA minor-groove. Namely, we calculated the root-mean-squared deviation (RMSD) of the intercalation-loop heavy atoms (P270-R281) for each microstate belonging to certain metastable state referenced to the corresponding IC, by firstly fitting the heavy atoms of four ribose groups (the interrogated bp and one adjacent bp). Then, we projected the MD conformations with the calculated RMSD < 3 Å onto the same two tICs given in Figure 2 (see Figure 3A). Intriguingly, we observe that S4, S5 and S6 prefer to sample the intercalation-loop conformations that resemble to the bp-interrogating form compared to others, indicating that as TDG approaches to the lesion site, the intercalation loop tends to penetrate deeper into the minor groove. This conclusion is further supported by the analyses that the intercalation loop in S5 has relatively smaller solvent accessible surface area (SASA) (see Figure 3B), suggesting that the loop invasion can profoundly exclude the solvents from the minor groove.

Remarkably, the DNA chain exhibits the largest bending angle (>20°) in S5 where TDG targets to the lesion site (see Figure 3C and Supplementary Figure S13A). Moreover, the presence of TDG can profoundly increase the possibility of the opening event for the G·T mispair (see Figure 3D and Supplementary Figure S13B), likely caused by the intrinsic instabilities of the mispair and the improper adjacent base stacking. In contrast, the non-specific complexes demonstrate distinct structural features in terms of the DNA conformation and bp dynamics. That is, the DNA chain bends at a range of ∼14–18° for the non-specific complexes (see Figure 3C). Moreover, the bps at the non-specific sites display almost no (S1, S2, S3, S4, S7 and S9) or moderate (S6 and S8) changes for the opening angle before and after TDG binding (see Supplementary Figure S14).

### The intercalation loop recognizes the lesion site by widening the minor groove and displaying a specific conformation

**Figure 4.**
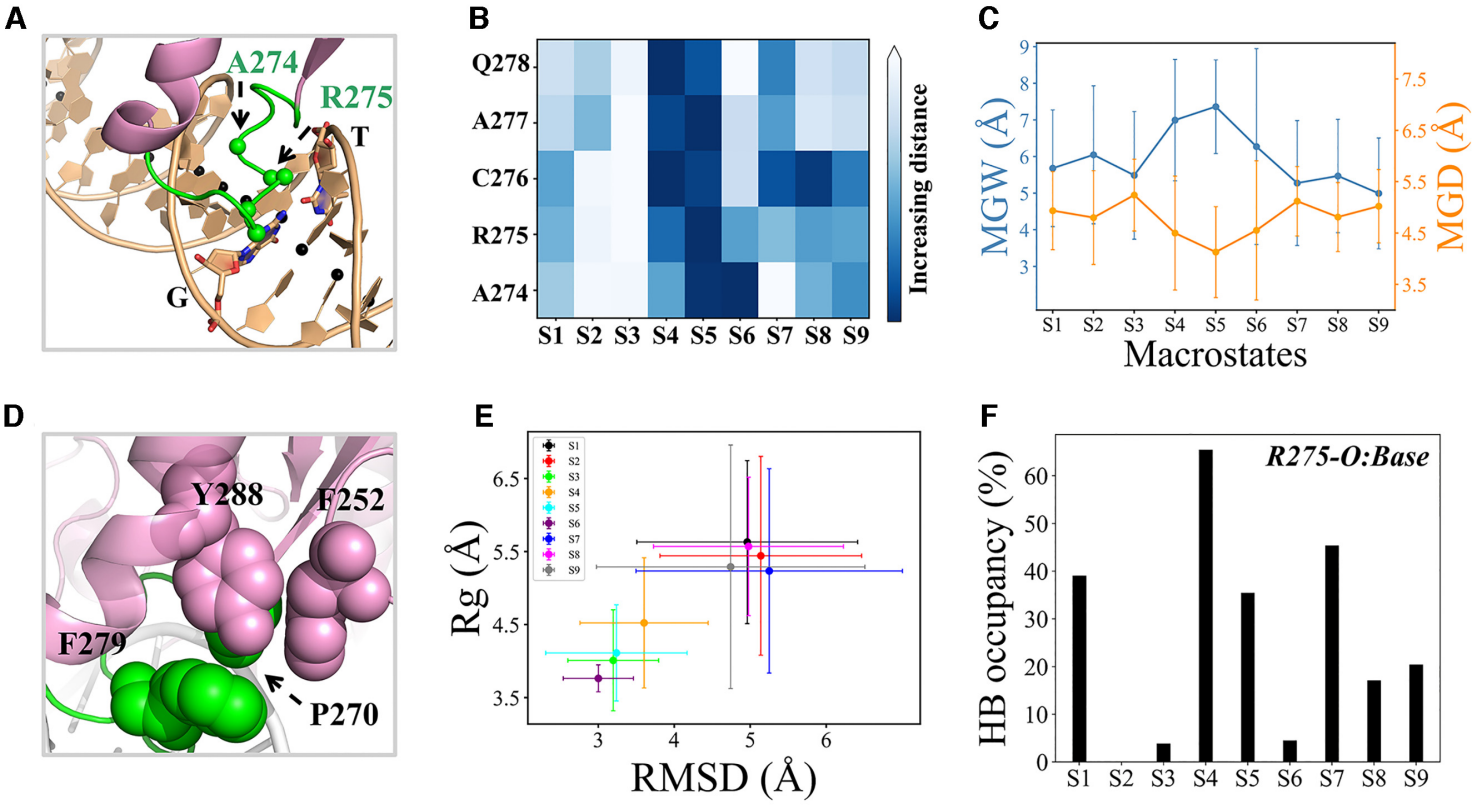
(**A**) Structural representation for calculating the interactions between the intercalation loop and nucleobases. The DNA chains are shown as orange ribbons; the COM of each bp are shown as black spheres; the C_α_ atoms of A274-Q278 are shown with green spheres; the G·T mispair is shown in orange sticks. (**B**) The minimum distance of each C_α_ atom of A274-Q278 to the COMs of bps. The average value for each metastable state is provided. (**C**) The minor groove width (MGW) and minor groove depth (MGD) at the G·T site for each macrostate. Refer to Supplementary Figure S13C for the structural illustration. (**D**) A hydrophobic core formed by P270, F279, Y288 and F252 (highlighted with sphere models) is critical for stabilizing the intercalation loop in a specific conformation. (**E**) The radius of gyration (Rg) of P270, F279, Y288 and F252 against the RMSD of the intercalation loop (P270-R281) for each metastable state. The RMSD was calculated by fitting the C_α_ atoms of TDG excluding all flexible loop regions, using the lesion-targeting TDG–DNA IC as the reference. (**F**) The HB occupancy between residue R275 mainchain and nucleobases for each macrostate. For the calculations of MGW, MGD and Rg, the mean value was obtained by averaging all the microstates belonging to the same macrostate, and the corresponding standard error was then calculated.

To further investigate the interaction networks between the intercalation loop and DNA nts, we measured the minimum distances between the C_α_ atom of five loop residues (A274-Q278) and the COMs of each bp (see Figure 4A). These residues are selected because they are all buried in the minor groove and have direct interactions with the nts, as observed in the crystal structures. The results show that in S4 and S5 the above loop residues are relatively close to the nucleobases compared to other states, especially for S5 (see Figure 4A and B). This is consistent with the above results that the intercalation loop in S5 is less solvent-exposed, suggesting solvent expulsions may take place as TDG approaches the lesion site in order to maximize its contacts with the interrogated bp (see Figure 3B). Meanwhile, insertion of the intercalation loop into the minor groove widens the minor-groove width (MGW) and induces a relatively shallow minor-groove depth (MGD) (see Figure 4C and Supplementary Figure S13C), which in turn increases the contact probabilities between the intercalation loop and the nucleobases.

More importantly, the structure of the intercalation loop in S4, S5 and S6 also closely resembles to the loop-conformation observed in the crystal structure (PDB id: 2rba), with an average RMSD difference <4 Å (see Figure 4E). It is also noteworthy that the crystal-like loop-conformation can also be observed in S3 (see Figure 4E), nevertheless, the interactions between the loop residues and the nucleobases are poorly formed (see Figure 4B). This result suggests that, during TDG sliding along DNA, the specific loop-conformation can also be transiently formed, but only when approaching the G·T mispair can the intercalation loop insert deeply into the minor groove by forming strong interactions with the nucleobases. On the other hand, the intercalation loop in other states all demonstrates non-specific conformations, with the key loop residues keeping relatively far away from the nucleobases (see Figures 4B and E). Notably, the key intercalated residue R275 is found to form one critical HB with the guanine group via its backbone C = O when TDG interrogates the G·C/C·G bps, i.e. for S1, S4, S5, S7 and S9, while this HB is destroyed when it binds with the A·T/T·A bps, i.e. for S2, S3 and S6 (see Figures 3A and 4F). Notably, the relatively high HB occupancy observed in S8 is in fact attributed to two adjacent G·C bps. The above results may provide the molecular explanations for the fact that TDG tends to target to the CpG-riched region (82,83).

Intriguingly, we find that in the specific loop-conformation, the loop-residue P270 and F279 prefer to form direct non-polar contacts with the residues F252 and Y288 from the surrounding TDG motifs (see Figure 4D). To examine the functional role of the above hydrophobic interactions, we calculated the radius of gyration (Rg) of the abovementioned four residues for different states. The results demonstrate a strong correlation between the calculated Rg and the conformation of the intercalation loop. That is, the specific loop-conformation tends to establish stable hydrophobic contacts with F252 and Y288 (see Figure 4E). In other words, the formation of the hydrophobic core contributes to maintaining the intercalation loop in a specific conformation. This provides a plausible explanation for the observation that S3, in which the intercalation loop exhibits a crystal-like conformation and poorly interacts with DNA, is less exposed to the solvents (see Figure 3B).

### Critical positively charged residues responsible for TDG translocation along DNA

**Figure 5.**
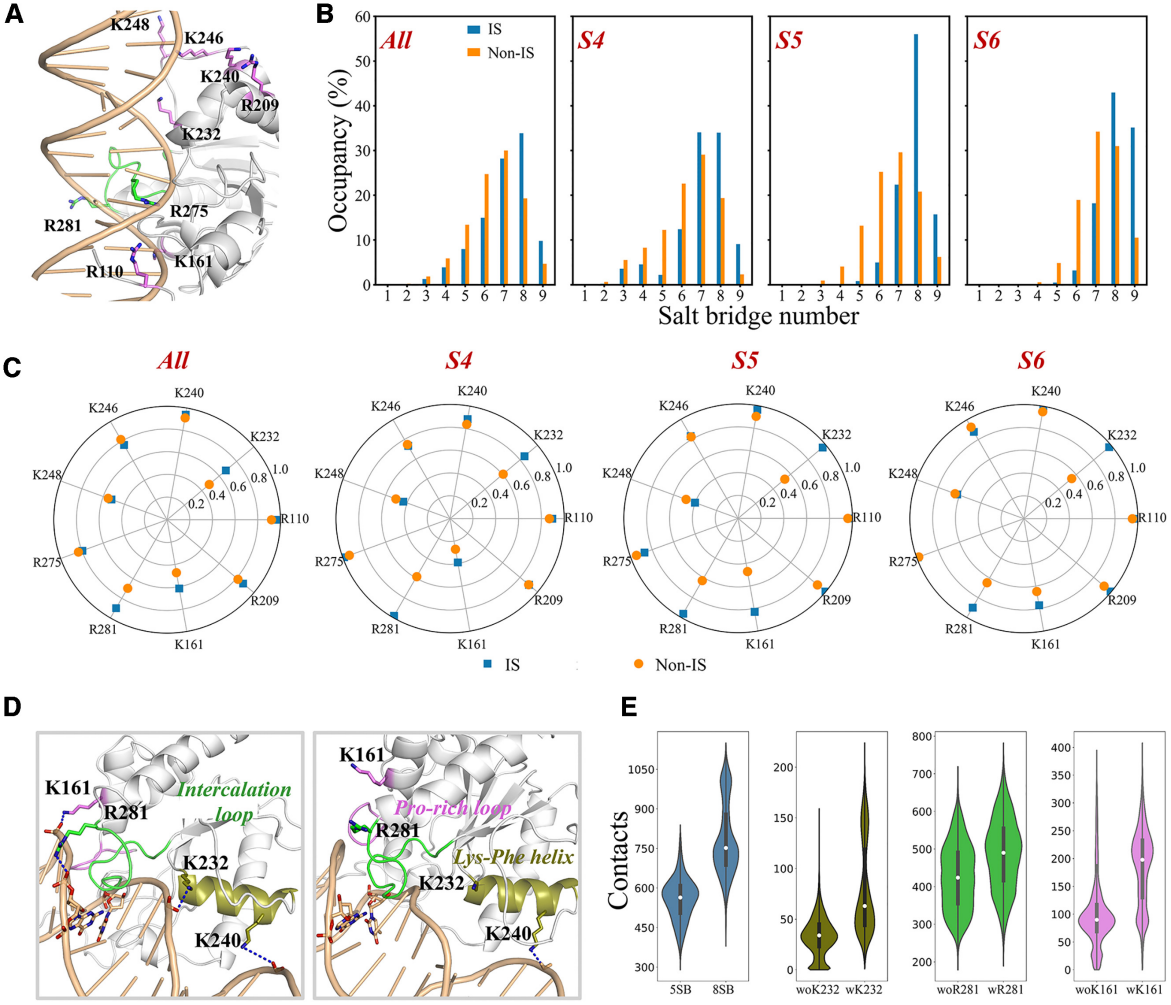
(**A**) Structural representation of nine positively charged TDG residues used for the salt-bridge interaction analyses between TDG and DNA. The nine residues are shown in green/pink sticks; DNA is shown in orange cartoon; TDG is shown in gray cartoon with the intercalation loop colored in green. (**B**) The occupancy of salt-bridge numbers for the interrogated and non-interrogated states (IS and non-IS) for the whole simulation dataset (All), S4, S5, and S6, respectively. One salt bridge interaction is defined using a minimum distance cutoff of 6 Å between the Lysine-NZ/Arginine-NH1/NH2 atoms and the O atoms of the DNA backbone. (**C**) The occupancy of the salt-bridge formed between each charged residue and the DNA backbones for the whole simulation dataset (All), S4, S5, and S6, respectively. (**D**) Representative S5 conformation that contains a total of eight (*left*) and five (*right*) salt bridges, respectively. The key salt bridges are represented by blue dashed lines. The key residues are shown in sticks. Three key structural motifs in TDG are colored by olive for the *Lys-Phe helix*, green for the *intercalation loop* and pink for the *Pro-rich loop*. (**E**) For S5, the salt bridge formation dictates the TDG binding with DNA. Violin plots of the contact number between three TDG motifs and DNA when concurrently forming eight (8SB) or five (5SB) salt bridges (left panel); in addition, the contacts between individual TDG motif with DNA, including the Lys-Phe helix (olive), intercalation loop (green), and Pro-rich loop (pink), are also calculated with and without the salt bridge formed via the corresponding charged residue (K232, R281 and K161, respectively).

Electrostatic interactions between DNA-binding proteins and DNA are considered to play essential roles in facilitating the protein binding and site-transfer (27,34,84). To examine the functional roles of the electrostatic interactions in the TDG sliding and lesion search, we chose nine positively charged TDG residues that can potentially form direct contacts with the DNA backbone, including K248, K246, K240, K232, R209, R275, R281, K161 and R110, and analyzed the salt-bridge interactions between each above residue and DNA backbones (see Figure 5A). To achieve this, for each metastable state, we defined two conformational spaces, comprising of the TDG conformations that near to each inspected bp (interrogated state, IS) and that transit between two adjacent bps (non-interrogated state, non-IS).

Then, from each metastable state, we randomly selected 10 000 TDG–DNA complexes from the above-defined IS and non-IS space, respectively (see Supplementary Figure S15). We next analyzed the salt-bridge interactions between TDG and the DNA backbone. For all nine metastable states, compared with non-IS, IS has higher probability to concurrently establish eight or nine salt bridges (see Figure 5B). We further pinpoint that R281, K161 and K232 are the key residues that dictate the difference of the salt-bridge interactions between IS and non-IS (see Figure 5C). In addition, we selected S4, S5 and S6 for more detailed analyses. The results show that the ISs for all the three states have higher tendency to form more salt-bridges compared to the corresponding non-ISs, especially for S5, in which the concurrent formation of eight and nine salt-bridges is almost doubled in IS (see Figure 5B). In consistent, R281, K161 and K232 are found to be the determinant residues to differentiate IS and non-IS, suggesting their significant roles in mediating the site-transfer of TDG between different bp sites (see Figure 5C).

Notably, the above identified three residues are located in/near to three different motifs, namely, the Lys-Phe helix (K232-F243), the intercalation loop (P270-R281), and the Pro-rich loop (Y152-N157) (see Figure 5D). From one representative S5 structure that contains 8 salt-bridges in the TDG–DNA interfaces, it is clear to see that R281 and K161 exert strong electrostatic interactions with the DNA backbone, thereby positioning the intercalation and Pro-rich loop in a proper conformation that penetrates into the minor groove and forms favorable contacts with the nucleobases (see the left panel in Figure 5D). Moreover, K232, together with K240, can form salt-bridge contacts with the DNA backbones by bridging two separate DNA strands, which potentially stabilize the Lys-Phe helical domain lying across the DNA major-groove. In contrast, the loss of the above three salt-bridge contacts profoundly destroys the interaction networks between TDG and DNA (see the right panel in Figure 5D), reflected from the significant decrease of the DNA contact number for corresponding structural motif (see Figure 5E). Taken together, K161, R281 and K232 likely serve as critical conformational switches as TDG transits between IS and non-IS, for the former, these residues prefer to bind with the DNA backbones, thereby anchoring the key DNA-recognition motifs of TDG in interrogated conformation.

### Determinations of the predominant state-to-state transitions within S4, S5 and S6 at microstate level

**Figure 6.**
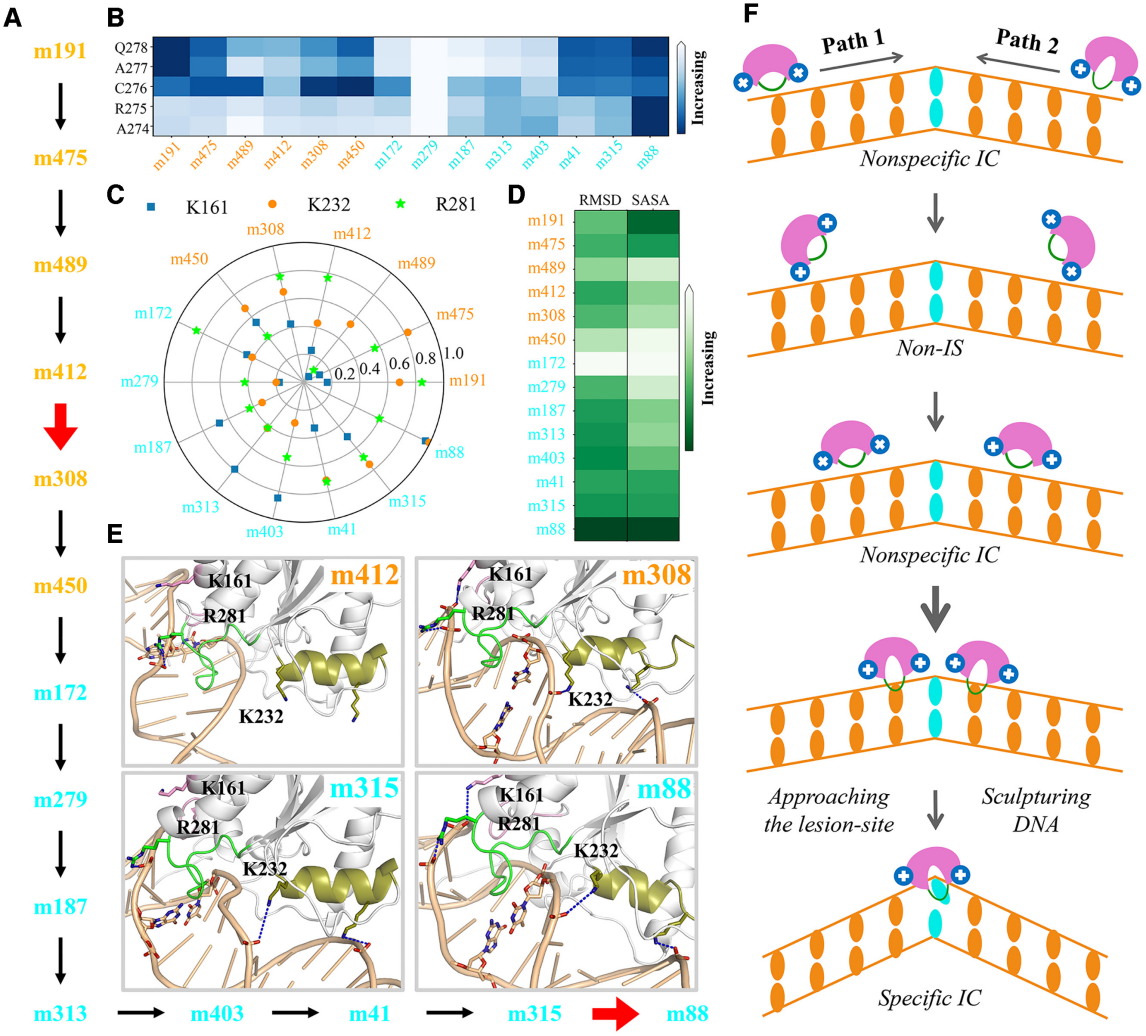
Determinations of the predominant microstate transition path during the S4→S5 transition by TPT. (**A**) The dominant state-to-state transition path from the non-specific IC in S4 (m191) to the specific IC in S5 (m88). The rate-limiting steps within S4 and S5 is highlighted with red thick arrows. (**B**) The minimum distance of each C_α_ atom of A274-Q278 to the COMs of bps. The average value for each microstate state is provided. (**C**) The occupancy of the salt-bridge formed with DNA backbones via K161, K232 and R281 for each microstate state. (**D**) RMSD and the SASA of the intercalation loop (P270-R281) for each microstate state. The RMSD was calculated by fitting the C_α_ atoms of TDG excluding all flexible loop regions, using the lesion-targeting TDG–DNA IC as the reference. (**E**) Representative structures of microstates involved in the rate-limiting steps shown in (A). Ref to Figure 5D for more details of the structural representations. (**F**) A schematic illustration for the rotation-coupled sliding dynamics of TDG along DNA minor-groove. The width of the arrow reflects the kinetic rates of the transitions (thicker arrow indicates slower transition).

To further pinpoint the specific microstate transition responsible for limiting the structural dynamic within each macrostate, we looked deep into our 500-state MSM that could provide us sufficient resolution to investigate more detailed state-to-state transitions at microstate level. In specific, we pinpointed the dominant path of two individual bp transitions that are involved in the last bp transition to target the G·T mispair, namely S4→S5 and S6→S5 (see Figure 2C). The dominant path of each above bp transition was chosen as the top transition path (with the largest net flux) determined by TPT. In details, for the S4→S5 transition, the dominant transition path from the non-specific IC in S4 (belonging to the microstate #191, or m191) to the specific IC in S5 (microstate m88) is determined as a path involving 14 microstates. The state-to-state transition is shown in Figure 6A, in which the rate-limiting step within S4 and S5 is m412→m308 and m315→m88, respectively. Further structural analyses reveal that both above two slow transitions can lead to deeper insertion of the intercalation loop into the minor-groove, reflected from the profound distance decrease between the loop-residue and DNA nucleobases (see Figure 6B). Particularly, the m315→m88 transition can result in an intercalation-loop conformation resembling the most to that observed in the lesion-targeting TDG–DNA IC (see Figure 6D). It is also noteworthy that the structural change of the intercalation-loop is tightly coupled with its SASA variations. That is, when TDG nears to the inspected bp, i.e. for m191 and m88, the intercalation-loop inserts deeply into the minor-groove by expelling the solvents; whereas when TDG transits between two adjacent sites, e.g. for m450 and m172, the intercalation-loop tends to expose to the solvents via adopting distinct conformations.

Moreover, we further examined how the above identified three positively charged residues might be involved in the above dominant path during the S4→S5 transition. We find that the rate-limiting step within S4 or S5 can both lead to more stable electrostatic interactions between TDG and DNA, which assist in anchoring the aforementioned key DNA-recognition motifs of TDG in interrogated conformation (see Figures 6C and 6E). Particularly, for both m412→m308 and m315→m88 transitions, two salt-bridges formed with DNA backbone via K161 and K232 are profoundly strengthened. Importantly, m88 exhibits the highest occupancy of the salt-bridge contacts via K161 and K232 among all microstates (see Figures 6C and 6E), consisting with the fact that m88 is the lesion-targeting IC.

For the S6→S5 transition, on the other hand, the dominant microstate transition path consists of 8 microstates (see Supplementary Figure S16A). Notably, only one state-to-state transition is observed within S6 (namely m218→m232), and the rate-limiting step in S5 is m315→m88, which is the same one as determined in the dominant path involved in the S4→S5 transition (compare Figure 6A and Supplementary Figure S16A). Again, m88 displays the most stable electrostatic interactions with DNA via K161 and K232 among all 8 microstates, thereby promoting its deep penetration into the minor groove (see Supplementary Figure S16B–D). Altogether, the microstate transition m315→m88 is responsible for limiting the TDG dynamics as it approaches to the target site. The structural dynamics of the intercalation-loop coupled with the desolvation process, together with the electrostatic interactions between TDG and DNA are found to be the key factors regulating the lesion-recognition of TDG.

## DISCUSSIONS

### Atomic-level revelation of the rotation-coupled sliding motion of TDG along DNA minor-groove during lesion search

Here, by performing extensive MD simulations combined with MSM construction, we studied the sliding dynamics of TDG within a 9-bp DNA segment for lesion search and recognition. Our MSM reveals nine metastable states (S1–S9), for each, TDG interrogates one certain bp. S5, in which TDG targets to the G·T mispair, has the highest thermodynamic stability among all states. Kinetically, TDG slides at a relatively faster rate when distant from the mismatched site, while the sliding slows down as TDG approaches the target-site (see Figure 6F). Before reaching to the lesion site, the intercalation loop in TDG can adopt a variety of non-specific conformations and inserts shallowly into the minor groove, shielded by the solvent waters. However, when TDG approaches the mispair site, the intercalation loop exhibits a specific conformation and invades deeply into the minor groove, establishing strong interactions with the nucleobases by expelling the solvent waters. Particularly for S5, the intercalation loop displays the deepest invasion into the minor groove, resulting in widening the minor groove, bending the DNA backbone, and partially opening up the mismatched bp. Moreover, we find the electrostatic interactions between TDG and DNA play key roles in mediating the site-transfer of TDG between adjacent bps (see Figure 6F). In particular, K161, K232 and R281 are responsible for mediating the TDG translocation along DNA, via strengthening/weakening the TDG–DNA interactions. Altogether, our work provides an atomic-level understanding of the 1D lesion-searching mechanism for TDG when approaching the target-site.

### TDG transits between distinct conformations during lesion search

The interplay between TDG and varied DNA sequence are one of our major focuses. We find that the intercalation loop in TDG can confer distinct conformations while locating at different bp sites. When TDG is far from the lesion site, the intercalation loop largely adopts a conformation different from the crystallographic structure and binds shallowly into the minor groove, resulting in a relatively fast transition rate between adjacent sites (i.e. few μs). However, as TDG approaches the lesion site, the intercalation loop prefers to adopt a specific conformation and penetrates deeply into the minor groove, which leads to a transient pausing that limits the overall sliding rate. Moreover, further analyses of the salt-bridge contacts between TDG and DNA reveal that TDG can establish more stable salt-bridge interactions with DNA backbone in IS compared with non-IS, which helps to anchor TDG in a proper orientation relative to DNA, especially for S5. Importantly, the site-transfer of TDG from one bp-site to the adjacent one requires to weaken the TDG–DNA interactions (by switching off several salt-bridges) in order to facilitate TDG translocation, and the rebinding of TDG to the next bp-site would re-establish the preceding salt-bridge interactions. Taken together, TDG undergoes profound structural changes while sliding along DNA by altering the intercalation-loop conformations and modulating the salt-bridge switches.

Notably, former experimental studies have also suggested that several DNA glycosylases, i.e. AAG (85), hOGG1 (24,30,86) and UDG (84,87), could undergo structural rearrangements while searching for the target sites along DNA. For example, NMR and biochemical studies by Stivers’ group have suggested that UDG can undergo profound conformational changes when sliding along DNA, thereby a two-state model, namely open and closed states, was proposed (84,87). That is, the open state corresponds to a state that weakly interacts with DNA, therefore, diffuses relatively faster along DNA. The closed state, on the other hand, can form stable contacts with DNA, resembling to the structures captured by crystallographic methods. Likewise, a two-state model was also proposed for hOGG1 using biochemical and Monte Carlo simulations (21,30). In addition, Drennan *et al.* captured two distinct DNA-binding conformations of AAG in crystal forms, one low-affinity state for non-specific binding and one high–affinity state for target recognition. In the latter, a more continuous and positively charge-riched AAG–DNA interface was observed (85). Considering the abovementioned DNA glycosylases belonging to different structural families, the structural changes of the repair enzyme involved in the sliding process along DNA are likely a general lesion-searching mechanism for a variety of DNA glycosylases.

### Identification of the key TDG residues responsible for the site-transfer and base-flipping

Our structural analyses reveal that three TDG residues, i.e. K161, K232 and R281, are involved in triggering the TDG translocation between adjacent bp sites by weakening the TDG–DNA interactions. Importantly, the formation of the above three salt-bridge interactions can substantially position three TDG structural motifs, including the Lys-Phe helix (K232-F243), the intercalation loop (P270-R281), and the Pro-rich loop (Y152-N157), in proper conformations that can form favorable interactions with DNA. Switching off the above three salt-bridges, however, results in weakening the TDG–DNA binding interfaces, which in turn facilitates TDG translocation. Structure-based sequence alignment of TDG, humun UNG (hUNG) and E. coli mispair-specific uracil glycosylase (MUG) demonstrate that K161 in TDG is highly conserved in hUNG and MUG (corresponding to Lys175 and Lys39, respectively, see Supplementary Figure S17A). K232, on the other hand, is conserved in MUG (namely Lys109), whereas is replaced by Ser247 in hUNG. Despite that, it is noteworthy that the Ser247 from hUNG, locating on the ‘Gly-Ser loop’, is highly conserved among the UNG family, thus it is highly probable that the Ser247 in hUNG might function similarly to K232 in TDG (see Supplementary Figure S17A-B). Finally, R281 in TDG, from the key intercalation loop, is found to be unconserved among the three glycosylases according to the sequence alignment. Nevertheless, one arginine residue, namely Arg276 in hUNG and Arg146 in MUG, can be observed close to the corresponding intercalation residue Leu272 (for hUNG) and Leu144 (for MUG), also positioning near to the DNA backbones (see Supplementary Figure S17B). Considering the intercalation region from above three DNA glycosylases adopt different structural folds, it can be expected that the abovementioned arginine residue might play similar roles in facilitating the protein transfer along DNA. Our work therefore warrants further experimental validations.

The intercalation residue R275 is considered to play an essential role in facilitating the base-flipping (36,58,88). Here, we find that the HBs formed between R275 and DNA sugar/backbones can significantly promote the opening of the G·T mispair (see Supplementary Figure S18A). In sharp contrast, for the non-specific sites, the bp opening angle has no apparent difference before and after TDG binding (see Supplementary Figure S14), although R275 can still form HBs with the DNA backbone/sugar (see Supplementary Figure S18B). This result emphasizes again the critical role of R275 in promoting the base-flipping of the target nucleobase. Consistently, by site-directed mutagenesis, former studies have found that the R275A or R275L mutations could both affect the extrusion of the mismatched T from the DNA duplex, these residue-substitution, based on our current study, can substantially undermine the TDG–DNA interactions (88). Moreover, we further measured to what extent the target nucleobase is flipped from the DNA helix stack in S5 by projecting all the S5 conformations onto two reaction coordinates: one pseudodihedral angle and the COM distance between the dT-O2 & -N3 atoms and its opposite dG-N1 & -O6 atoms (see the definitions in Supplementary Figure S19A). The results show that comparing to the completely base-flipped conformation that exhibits a pseudodihedral of ∼–150° and bp distance of ∼16 Å (highlighted with magenta circle in Supplementary Figure S19B), all the S5 conformations remain far from the fully extruded state, as highlighted by one representative S5 conformation (see Supplementary Figure S19B, C). Notably, our unbiased MD simulations are up to 100 ns each, which is still too short to capture the complete base-flipping dynamics that was previously estimated to occur at a timescale of tens of μs (58).

## Supplementary Material

gkaa1252_Supplemental_File

## Notes

Present address: Lin-Tai Da, Key Laboratory of Systems Biomedicine (Ministry of Education), Shanghai Center for Systems Biomedicine, Shanghai Jiao Tong University, 800 Dongchuan Road, Shanghai 200240, China.
