# Supplementary material for: Atomic resolution of short-range sliding dynamics of thymine DNA glycosylase along DNA minor-groove for lesion recognition
Source: Nucleic Acids Res. 2021 Jan 19;49(3):1278–93. doi: 10.1093/nar/gkaa1252 (PMC7897493; doi:10.1093/nar/gkaa1252)
Supplement: gkaa1252_Supplemental_File [file gkaa1252_supplemental_file.pdf]

# Supplementary Information

# Atomic Resolution of Short-range Sliding Dynamics of Thymine DNA Glycosylase along DNA Minor-groove for Lesion Recognition

*Jiaqi Tian, Lingyan Wang and Lin-Tai Da*[*]

Key Laboratory of Systems Biomedicine (Ministry of Education), Shanghai Center for Systems Biomedicine, Shanghai Jiao Tong University, 800 Dongchuan Road, Shanghai 200240, China.

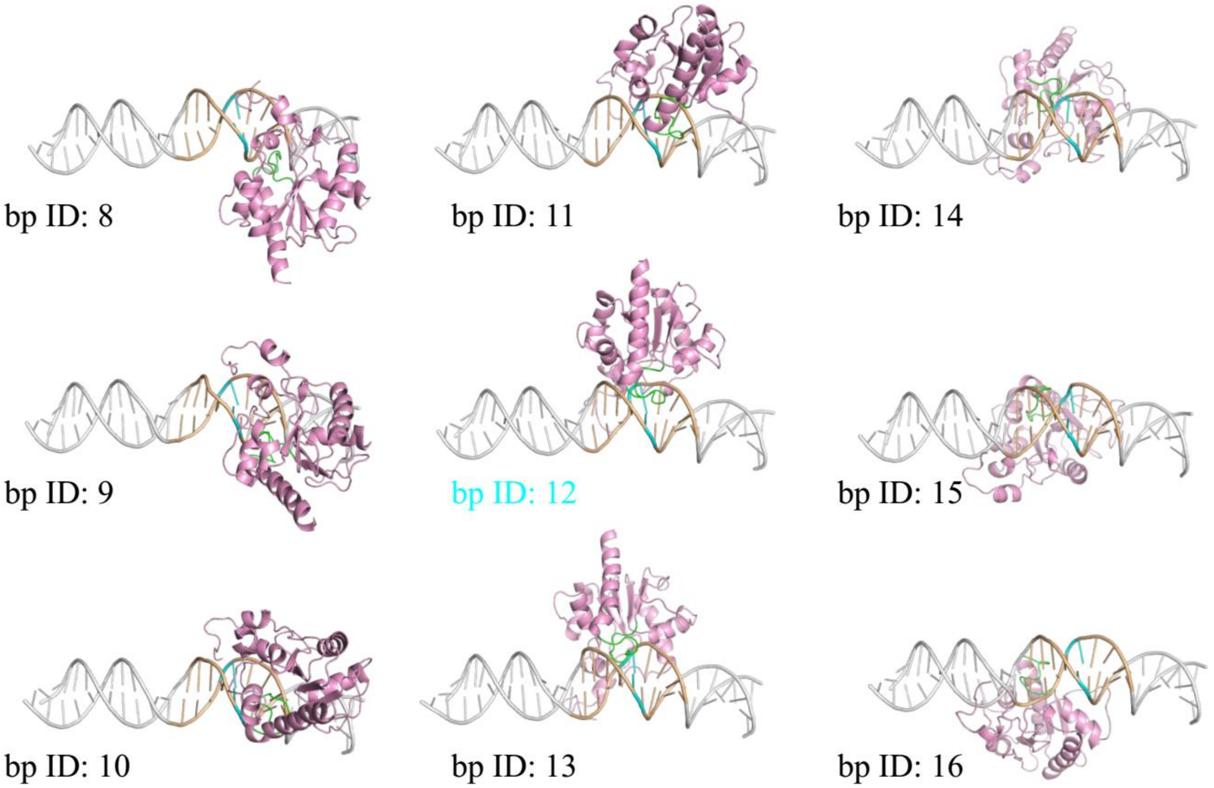


**Figure S1.** The 9 modeled TDG-DNA ICs, with TDG interrogating nine consecutive bp sites (from bps 8 to 16). Refer to main text Figure 1A for more details.

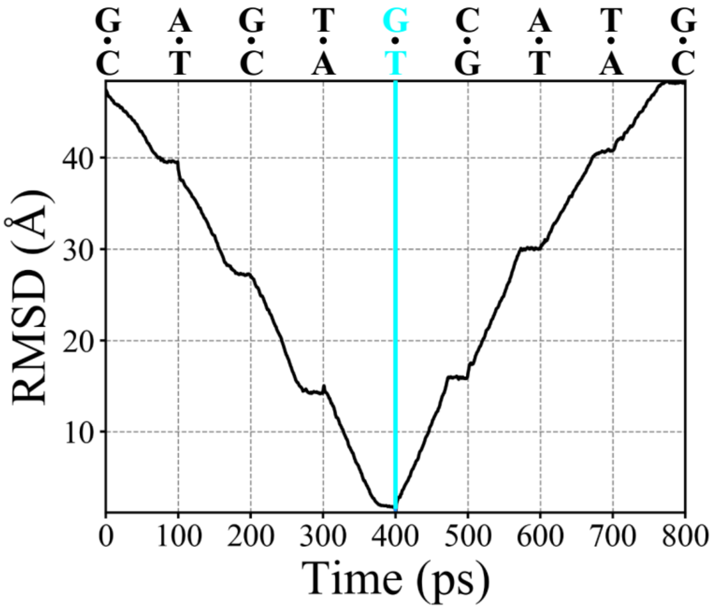


**Figure S2.** RMSDs of the TDG $C_\alpha$-atoms during the TMD simulations in the reverse direction (from bps 16 to 8), with respect to the lesion-targeting IC (at bp 12).

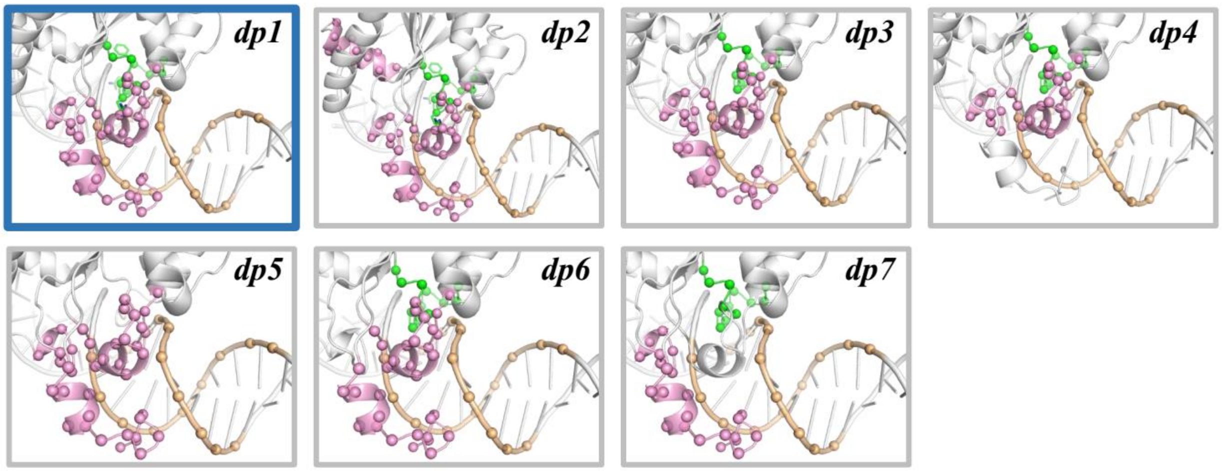


**Figure S3.** Seven different sets of distance pairs were designed and used for the tICA projection, namely *dp1-7*. The *dp1* includes the P atoms of one DNA segment (bps 8-16, shown in orange spheres); $C_\alpha$ atoms of four TDG motifs that can directly contact with DNA (namely, K107-K122, P141-H158, T196-D202, and P270-A282, shown in pink/green spheres), and side-chain heavy atoms of TDG residues R275, Q278, and F279 (green sticks). In addition, the *dp2-7* were designed to evaluate whether inclusion or exclusion of any TDG motif might impose any influence on the slowest implied timescale. In *dp2,* the TDG motif K232-F243 was included; in *dp3*, three TDG residues R275, Q278, and F279 were excluded; in *dp4-7*, each above DNA-binding motif was excluded.

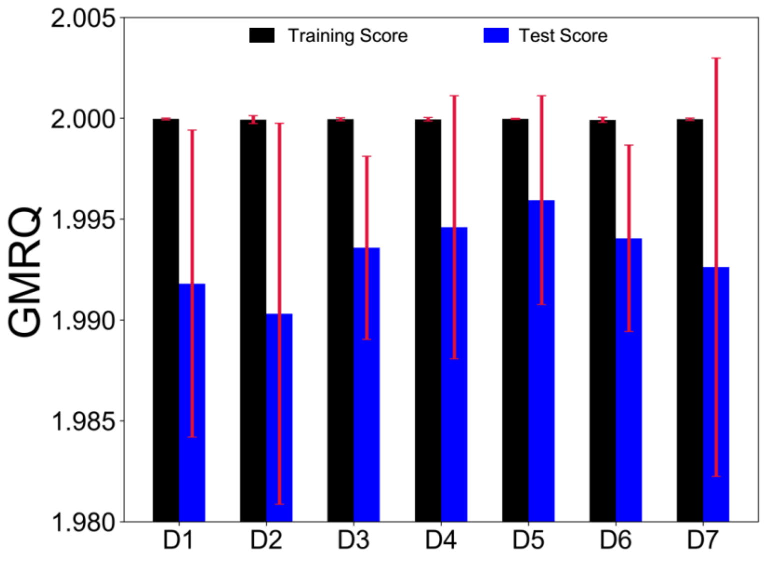


**Figure S4.** GMRQ validation for different sets of input distance pairs. The scores for both training and testing datasets are provided.

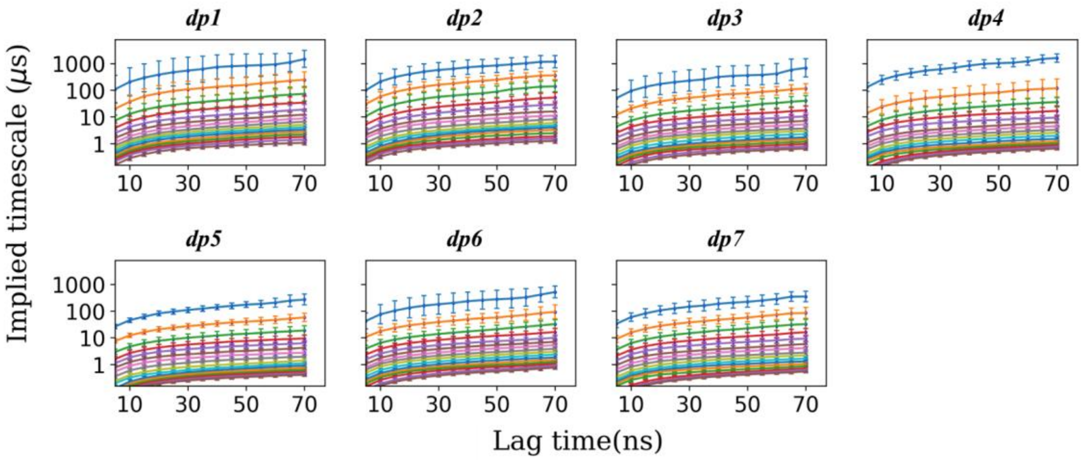


**Figure S5.** The implied timescale plots as a function of lag-time for the MSMs constructed by tICA using the input distance pairs shown in Figure S3. For each set, an MSM was constructed by projecting the MD conformations onto 4 slowest tICs followed by *K-centers* clustering to produce 500 states. The correlation lag time for tICA was chosen as 20 ns.

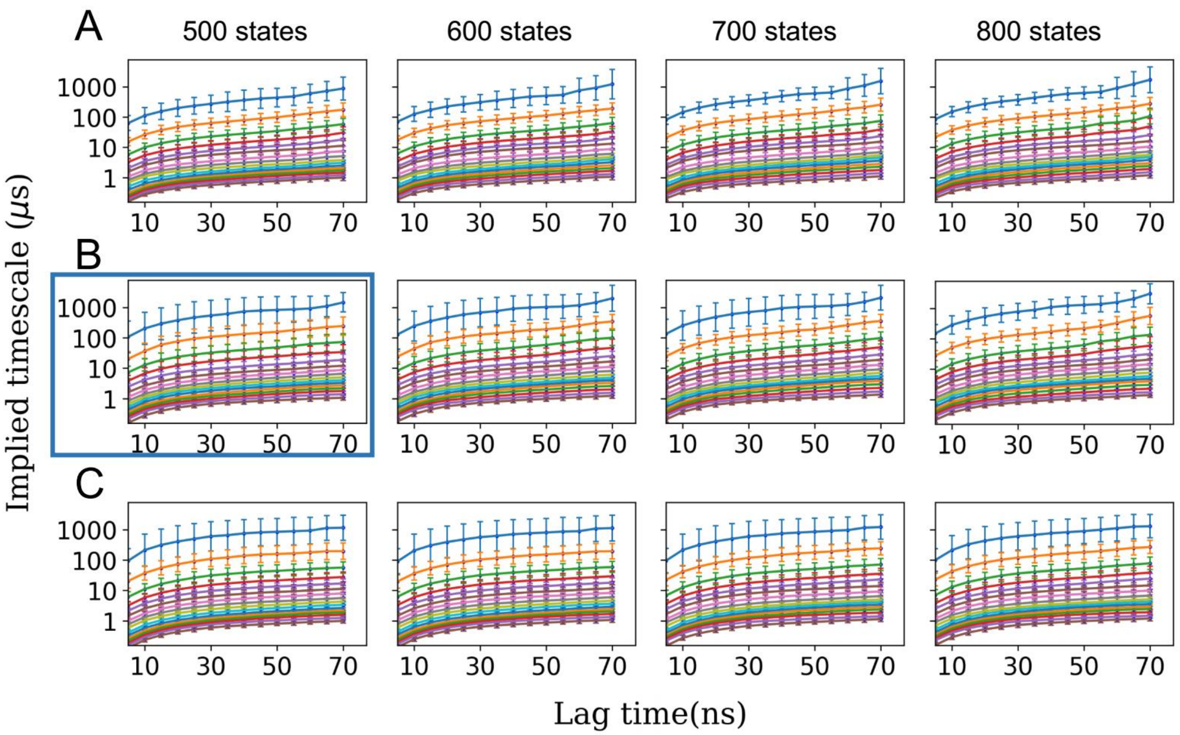


**Figure S6.** The implied timescale plots as a function of lag-time for the MSMs constructed by projections of MD conformations onto four slowest tICs followed by the *K-centers* clustering. From the top to bottom row, results from tICA with different correlation lag times (10 ns, 20 ns and 30 ns, respectively) are displayed. Under each correlation lag time, different number of microstates (500, 600, 700 and 800, respectively) are used for comparison. We finally chose the state number of 500, tICA correlation lag-time of 20 ns and top four tICs for the final MSM construction (in blue frame).

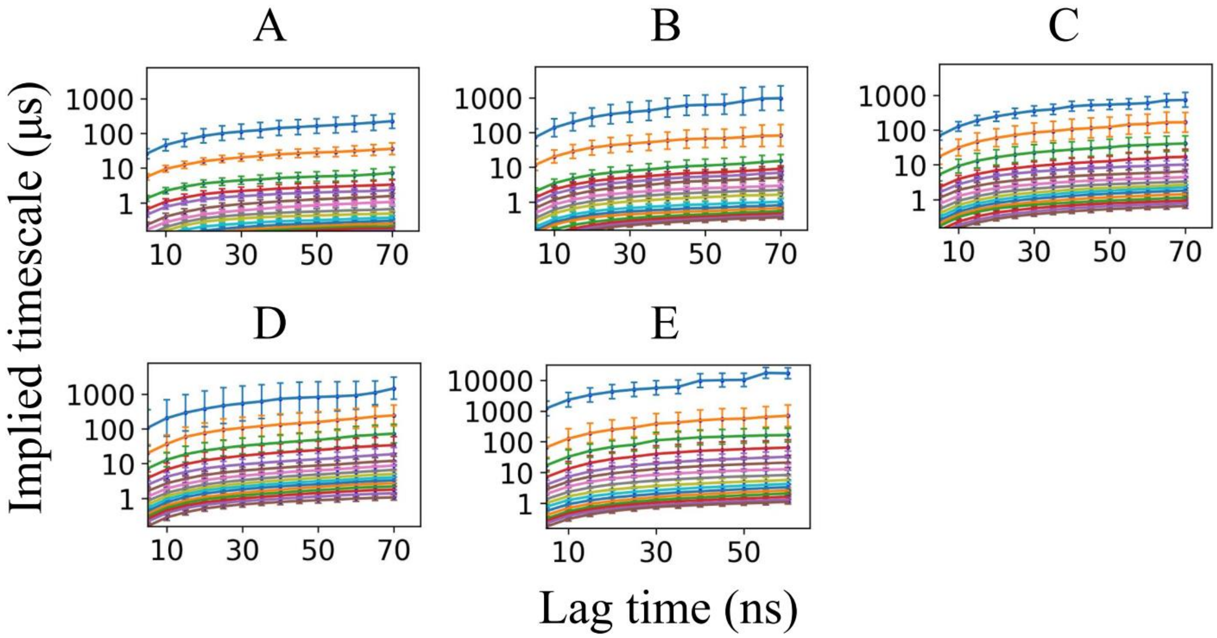


**Figure S7.** The implied timescale plots as a function of lag-time for the MSMs constructed by varying the tICs: (A) tIC1; (B) tIC1-2; (C) tIC1-3; (D) tIC1-4; (E) tIC1-5. The correlation lag time for tICA was chosen as 20 ns.

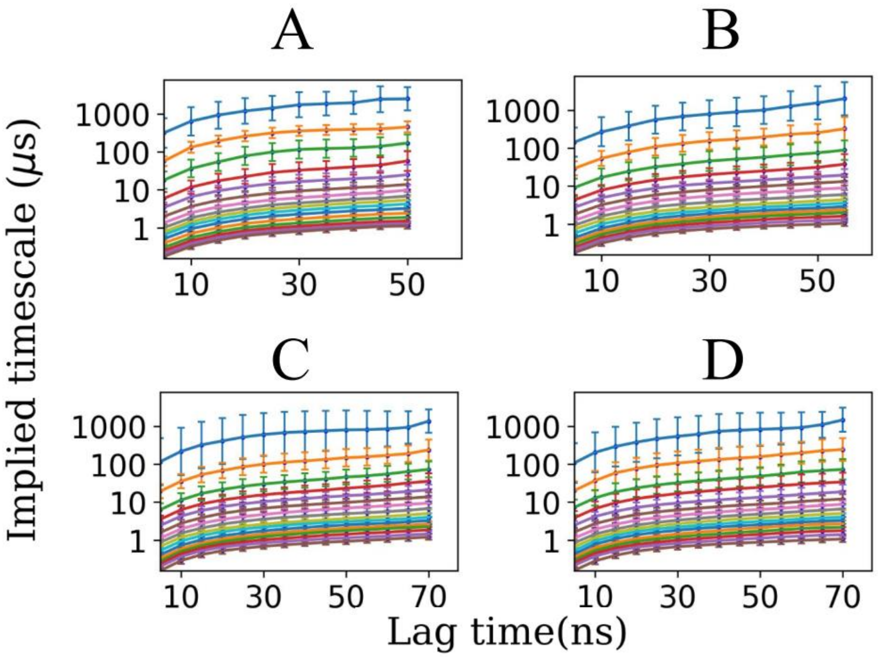


**Figure S8.** The plots of implied timescale against different lag time for varying aggregated simulation time: (A) 18 μs (70 ns × 252); (B) 20 μs (80 ns × 252); (C) 23 μs (90 ns × 252); (D) 25 μs (100 ns × 252).

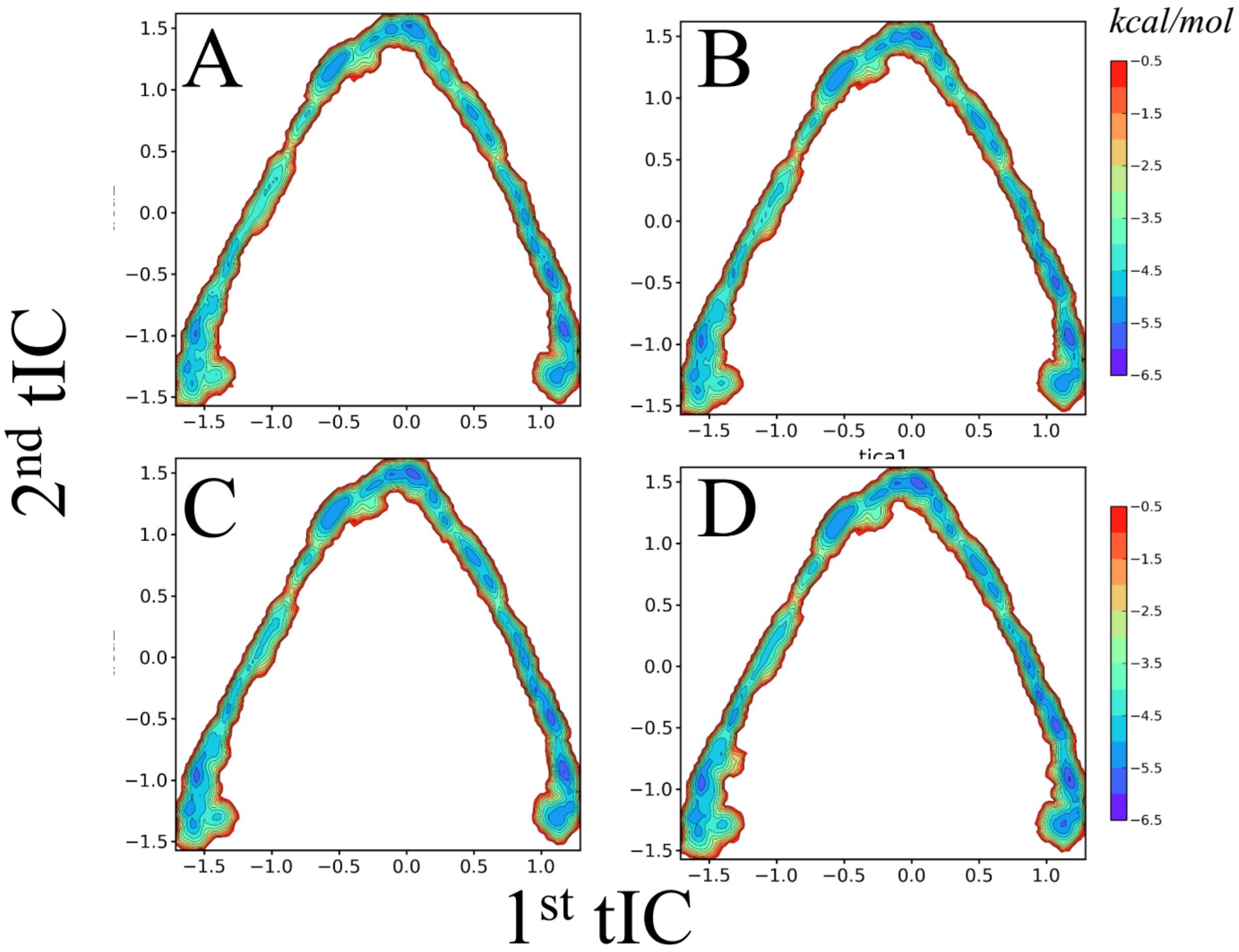


**Figure S9.** Free energy projections of different simulation datasets onto the top two tICs, with varied aggregated simulation time of (A) 18 μs (70 ns × 252); (B) 20 μs (80 ns × 252); (C) 23 μs (90 ns × 252); (D) 25 μs (100 ns × 252), respectively.

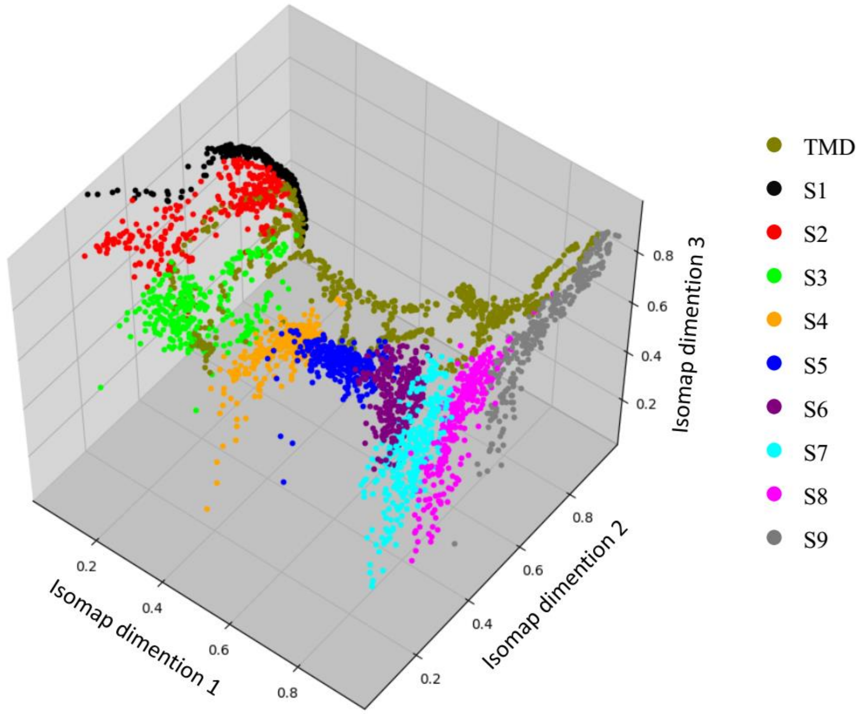


**Figure S10.** Projection of the macrostates identified from MSM and the TMD conformations onto the top three eigenvectors obtained by the Isomap analysis. For each macrostate, 300 representative conformations were chosen, and colored individually. In addition, the TMD conformations are also mapped onto the same phase space.

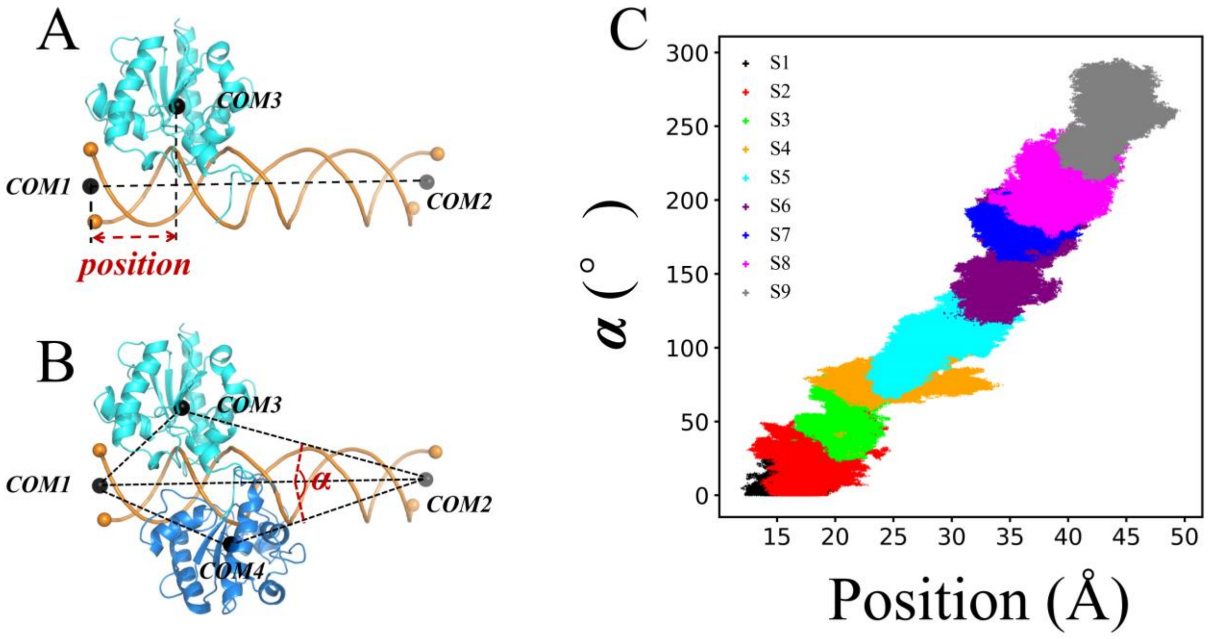


**Figure S11.** Strong correlation between the longitudinal and rotational motions of TDG during the sliding process was observed. (A) The longitudinal motion is described as the projected position of the COM of TDG $C_\alpha$ atoms onto a vector connecting the COM of two terminal P atoms from each DNA end (*COM1* and *COM2*), and the distance (or position) between the projected point and the *COM1* was calculated. (B) The rotational motion is described as a rotation angle ($\alpha$) defined as a dihedral angle formed by four COMs: *COM1* and *COM2* are the same as that defined in A; *COM3* is the COM of TDG $C_\alpha$ atoms (shown in cyan) from the initial IC model where TDG integrates at the bp-8 site; *COM4* is the COM of TDG $C_\alpha$ atoms (shown in marine) from each MD snapshot. (C) The scatter plot of the above defined projected position vs $\alpha$ for all MD conformations, with each metastable state labeled.

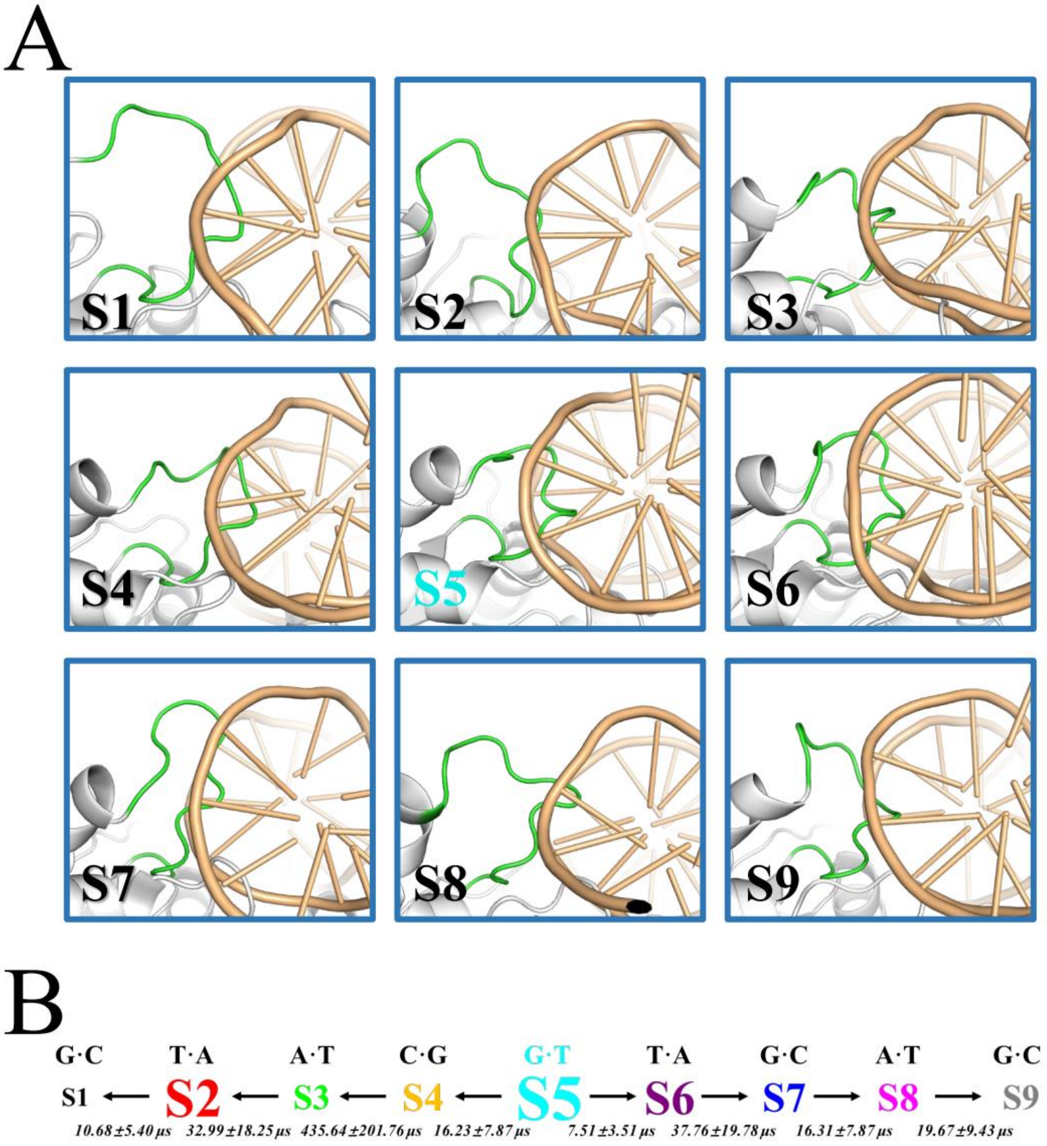


**Figure S12.** (A) Top views of the representative conformations for each metastable state. The same structures from the main text Figure 3A are used here. (B) The MFPTs for the backward inter-state transitions.

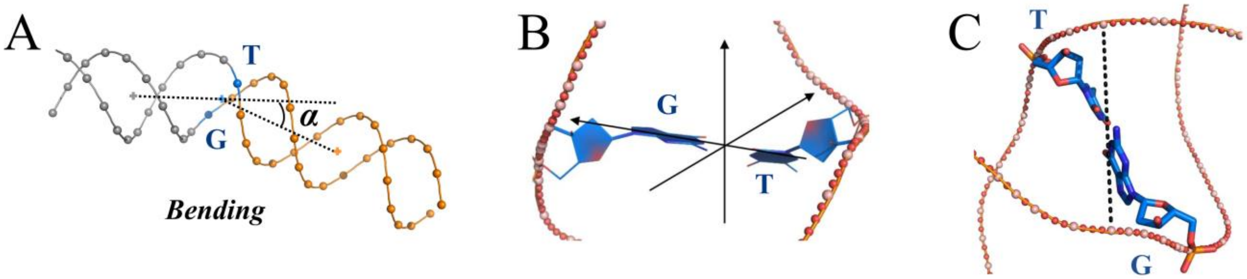


**Figure S13.** (A) Structural illustration of the DNA bending angle *α*. The DNA bending angle is defined as the angle between two vectors that are determined by three center of masses (COMs). The first (gray dot) is the COM of the P atoms (gray spheres) from bp1 to bp11; the second (blue dot) is the COM of the P atoms (blue spheres) of bp12; the third (orange dot) is the COM of the P atoms (orange spheres) from bp13 to bp28. (B) Structural illustration of the opening angle for certain bp. (C) Structural illustration of the minor groove width (MGW) for the G·T mispair.

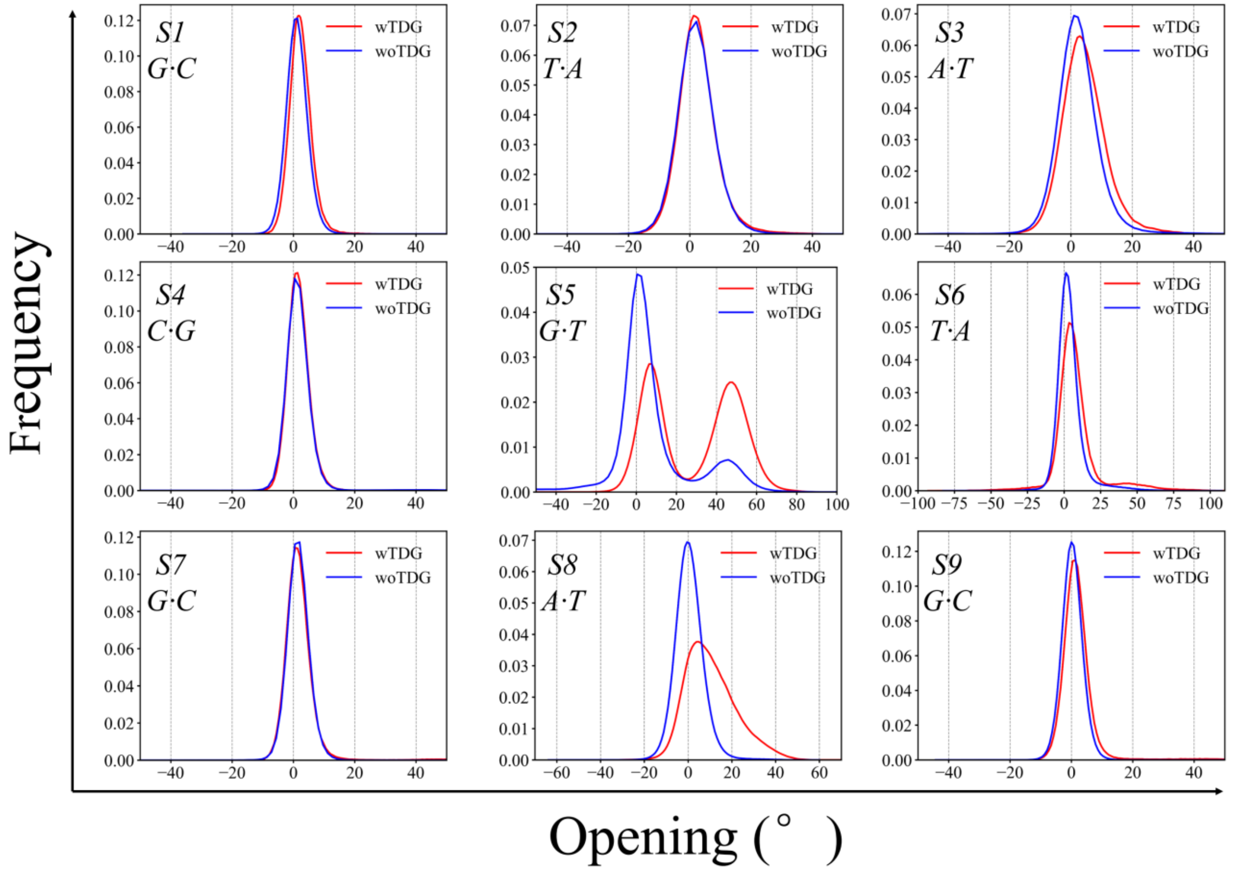


**Figure S14.** Distribution of the opening angle for the interrogated bp of each metastable state when TDG targets to the particular site (wTDG, red line) or other sites (woTDG, blue line). The opening angles are calculated using the Curves+ program (1).

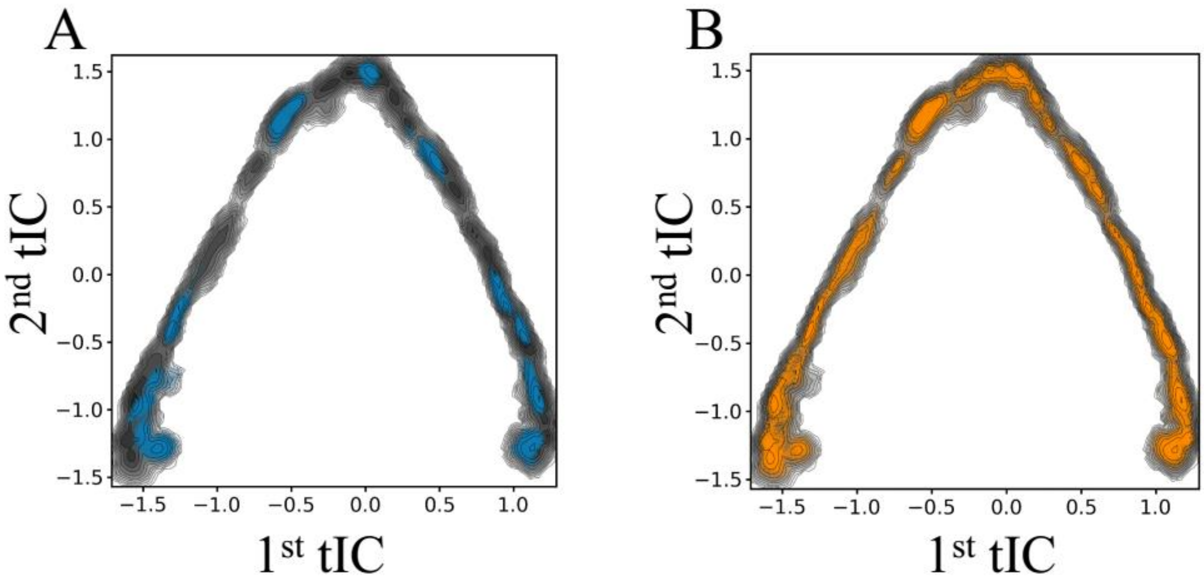


**Figure S15.** The scatter plots of representative MD conformations selected for salt bridge calculations mapped onto the slowest two tICs, (A) for interrogated state (IS, in blue), (B) for non-interrogated state (non-IS, in orange).

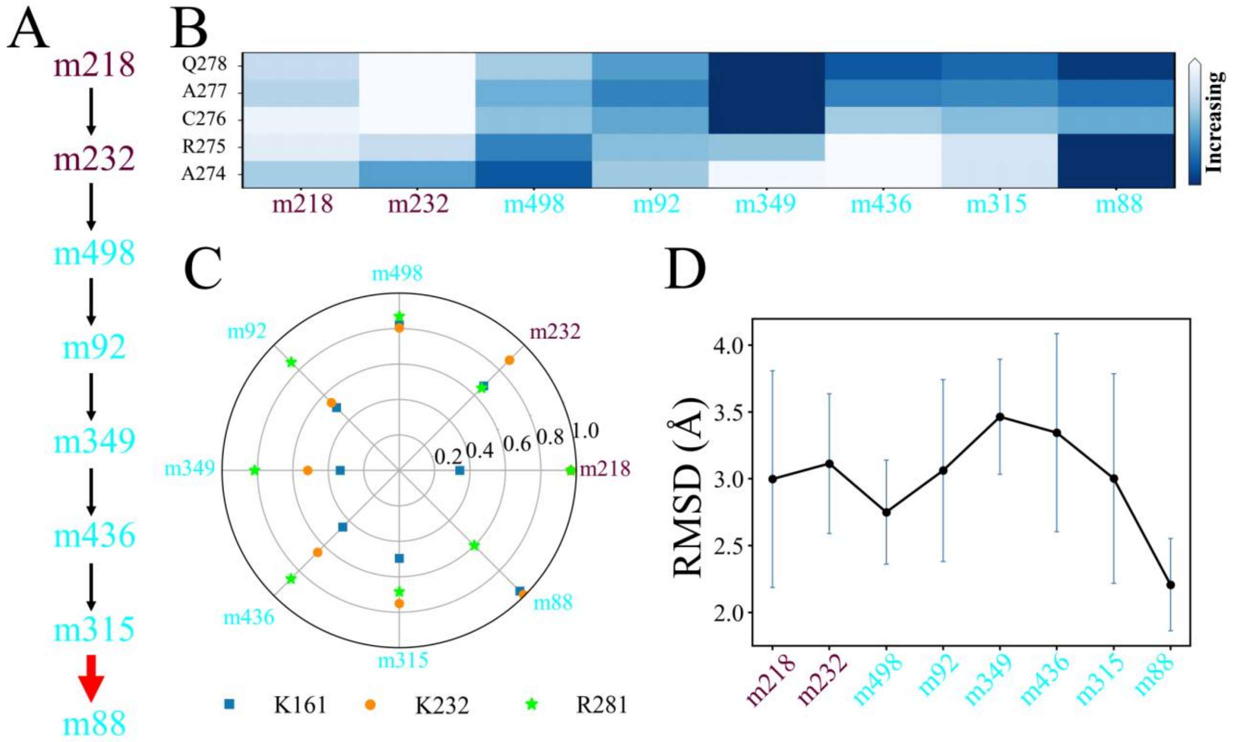


**Figure S16.** Determinations of the predominant microstate transition path during the S6→S5 transition by TPT. (A) The dominant state-to-state transition path from the non-specific IC in S6 (m218) to the specific IC in S5 (m88). The rate-limiting step within S5 is highlighted with red thick arrow. (B) The minimum distance of each $C_\alpha$ atom of A274-Q278 to the COMs of bps. The average value for each microstate state is provided. (C) The occupancy of the salt-bridge formed with DNA backbones via K161, K232 and R281 for each microstate state. (D) RMSD plot of the intercalation loop (P270-R281) for each microstate state. The RMSD was calculated by fitting the $C_\alpha$ atoms of TDG excluding all flexible loop regions, using the lesion-targeting TDG-DNA IC as the reference.

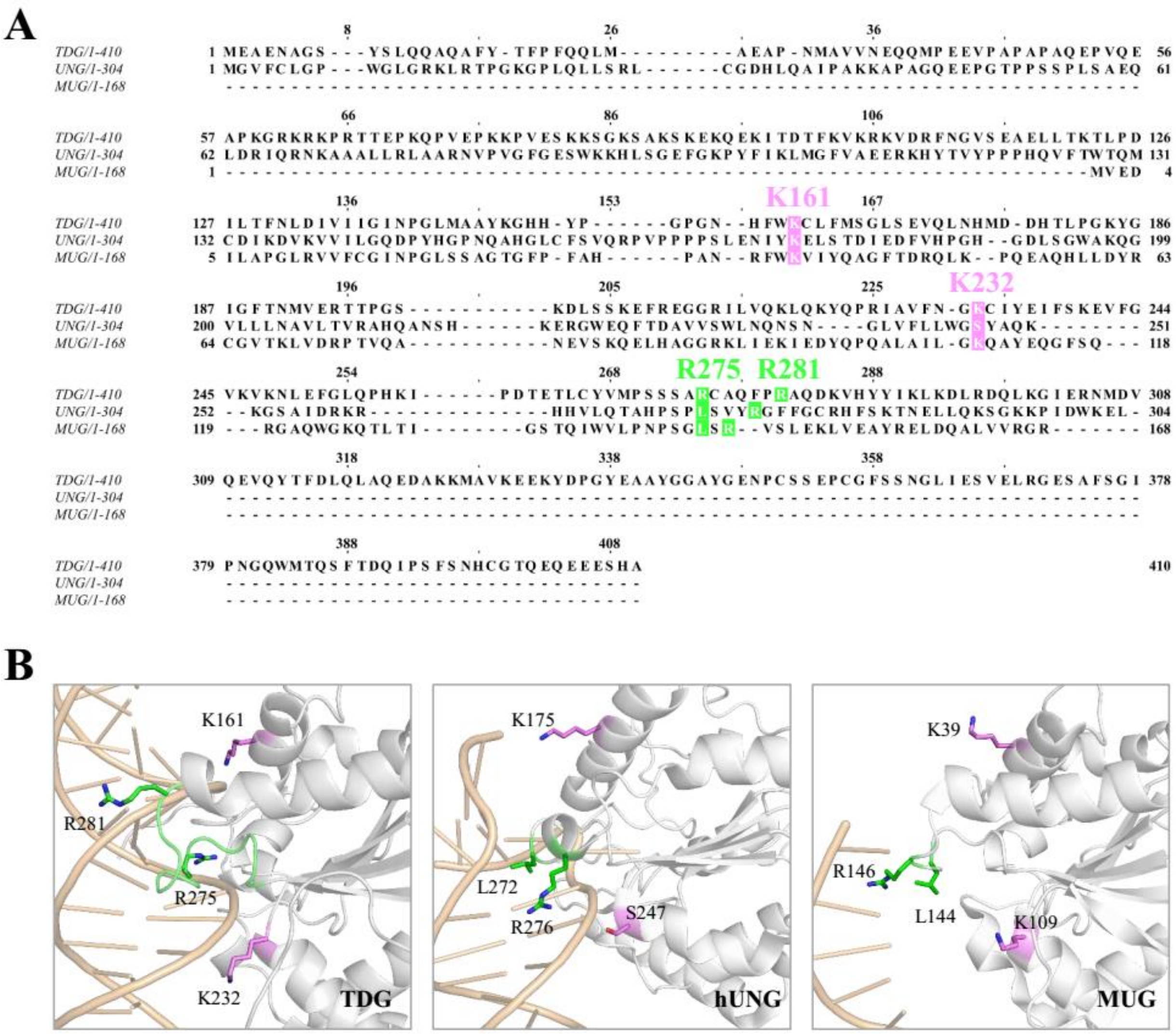


**Figure S17.** (A) Sequence alignment of TDG, hUNG, and MUG. TDG residues K161, K232, R275, and R281 are highlighted in green/purple. (B) Structural illustrations of three DNA glycosylases, with four key residues in each system highlighted with green/purple stick models. In particular, the intercalation residues, namely R275 in TDG, L272 in hUNG, and L144 in MUG, are colored in green. The hUNG and MUG sequences are derived from UniProtKB P13051 and P0A9H1, and the structures are created based on the PDB structures 1emh and 1mwj.

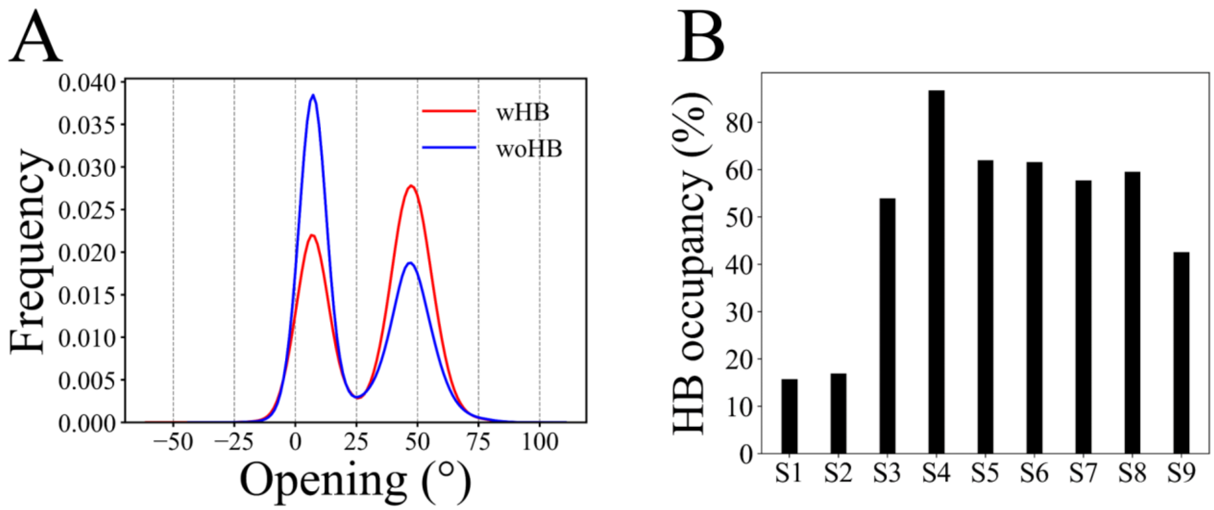


**Figure S18.** (A) In S5, the distribution of the opening angle for the G·T mispair with (red line) and without (blue line) the HB between the R275 sidechain and sugar atoms (O3' and O5') & DNA backbone atoms. (B) The above HB occupancy for each macrostate.

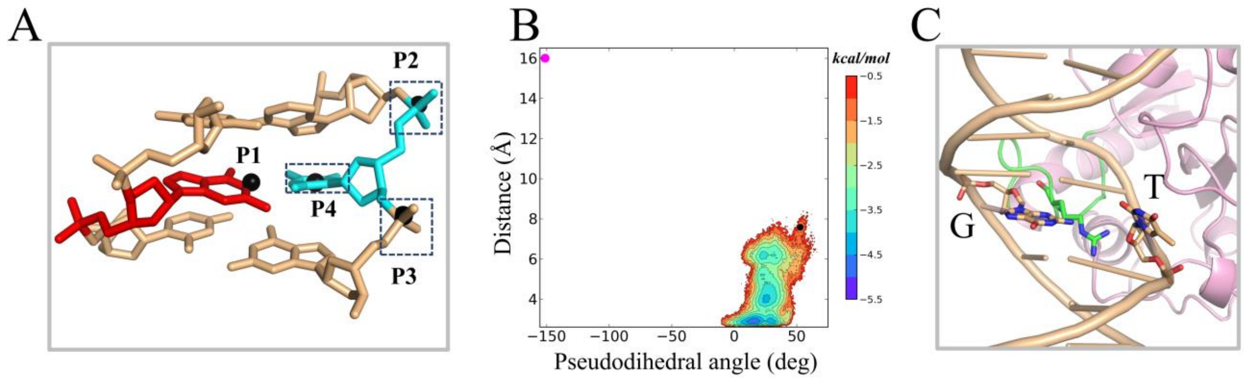


**Figure S19.** (A) Four points, P1-P4, are defined to calculate the pseudodihedral for the base-flipping process. P1 is the center of mass (COM) of the two base pairs adjacent to the mismatched bp (in orange); P2 and P3 are the COMs of two phosphate groups connecting to the flipped base, respectively; P4 is the COM of the six-member pyrimidine ring of the mismatched dT. The four COMS are shown in black spheres. (B) Free energy profile of the MD conformations projected onto two reaction coordinates: the pseudodihedral defined in (A) and the COM distance between the dT nt (O2 and N3 atoms) and its opposite dG nt (N1 and O6 atoms). The fully flipped nucleobase from one crystal structure of TDG-DNA complex (pdb ID: 5hf7) (magenta point), and one partially flipped base from S5 (black point) are also projected onto the same reaction coordinates. (C) One representative TDG-DNA conformation from the S5 state where the target nucleobase is partially flipped.

## RERERENCE

1. Blanchet, C., Pasi, M., Zakrzewska, K. and Lavery, R. (2011) CURVES+ web server for analyzing and visualizing the helical, backbone and groove parameters of nucleic acid structures. *Nucleic Acids Res*, **39**, W68-W73.
